# Kinetochore life histories reveal an Aurora-B-dependent error correction mechanism in anaphase

**DOI:** 10.1016/j.devcel.2021.10.007

**Published:** 2021-11-22

**Authors:** Onur Sen, Jonathan U. Harrison, Nigel J. Burroughs, Andrew D. McAinsh

**Affiliations:** 1 Centre for Mechanochemical Cell Biology, University of Warwick, Coventry, UK; 2 Division of Biomedical Sciences, Warwick Medical School, University of Warwick, Coventry, UK; 3 University Hospital Coventry and Warwickshire NHS Trust, Coventry, UK; 4 Mathematics Institute and Zeeman Institute, University of Warwick, Coventry, UK

**Keywords:** chromosome segregation, kinetochore, aneuploidy, lattice light-sheet microscope, computational analysis, error correction, Aurora B

## Abstract

Chromosome mis-segregation during mitosis leads to aneuploidy, which is a hallmark of cancer and linked to cancer genome evolution. Errors can manifest as “lagging chromosomes” in anaphase, although their mechanistic origins and likelihood of correction are incompletely understood. Here, we combine lattice light-sheet microscopy, endogenous protein labeling, and computational analysis to define the life history of >10^4^ kinetochores. By defining the “laziness” of kinetochores in anaphase, we reveal that chromosomes are at a considerable risk of mis-segregation. We show that the majority of lazy kinetochores are corrected rapidly in anaphase by Aurora B; if uncorrected, they result in a higher rate of micronuclei formation. Quantitative analyses of the kinetochore life histories reveal a dynamic signature of metaphase kinetochore oscillations that forecasts their anaphase fate. We propose that in diploid human cells chromosome segregation is fundamentally error prone, with an additional layer of anaphase error correction required for stable karyotype propagation.

## Highlights

• Chromosomes are at a considerable and continual risk of mis-segregation
• Metaphase dynamics forecast lazy kinetochores and their correction in anaphase
• Aurora B spindle midzone gradient enables phosphorylation of kinetochore substrates
• Results suggest midzone originated Aurora B activity destabilizes erroneous attachments

## Graphical abstract

**Figure:**
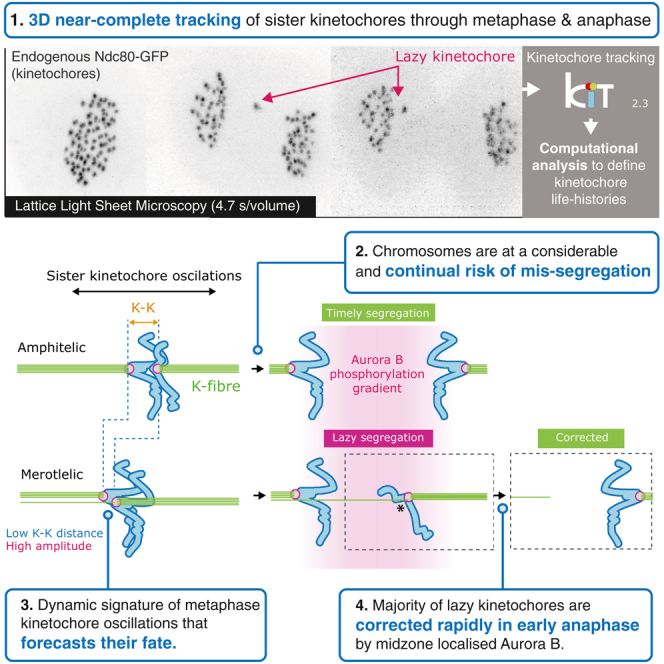

## Teaser

By tracking human kinetochores from prometaphase to late anaphase, Sen, Harrison et al. reveal that mitotic chromosomes are at a greater risk of mis-segregation than previously appreciated. They uncover an anaphase error correction mechanism that combats this mis-segregation and show that kinetochore behavior before anaphase can predict future segregation outcome.

## Introduction

Error-free chromosome segregation is a key task during mitosis and is crucial for maintaining the correct number of diploid chromosomes in a cell. Errors in chromosome segregation can lead to aneuploidy, the deviation in chromosome number from the diploid state. Such changes to the karyotype are associated with cancer progression, developmental disorders, and aging (Chunduri and Storchová, 2019; Nagaoka et al., 2012; Vasudevan et al., 2021). These pathologies are the consequence of gene dosage changes and/or proteomic stress (Sheltzer and Amon, 2011; Smith and Sheltzer, 2018). Mis-segregated whole chromosomes can also give rise to micronuclei that are distinct from the main daughter cell nuclei. The micronucleus can be a site of further mutational processes due to aberrant replication of the physically isolated chromosome, leading to chromothripsis, which is characterized by extensive genomic rearrangements (Stephens et al., 2011; Zhang et al., 2015). Similar to whole chromosome aneuploidy, chromothripsis is also associated with the evolution of human disease processes.

The chromosome mis-segregation rate in human cells is ∼1% (Santaguida and Amon, 2015). To ensure high-fidelity chromosome segregation, error correction mechanisms detect and destabilize improper attachments, including syntelics (sister kinetochore attached to one pole) and merotelics (one, or both, sister kinetochores forming attachments to both spindle poles; Biggins and Murray, 2001; Cheeseman et al., 2006; Liu et al., 2009). Through trial and error, sister kinetochores can achieve amphitelic attachments (both sisters attached to opposite poles) that are stable and compatible with accurate chromatid segregation (Gregan et al., 2011). The spindle assembly checkpoint (SAC) provides a further layer of protection by delaying anaphase onset when kinetochores are in an unattached state (also the outcome from error correction of syntelics) (Lara-Gonzalez et al., 2012; London and Biggins, 2014). However, merotelic attachments are invisible to the SAC and are thus a major source of aneuploidy (Cimini et al., 2001). This is likely a consequence of such kinetochores having sufficient microtubule occupancy and tension to satisfy the SAC (Cimini et al., 2001). Indeed, this can even cause stretching of the merotelically attached kinetochore due to pulling forces toward opposite poles (Cimini et al., 2001; Cojoc et al., 2016). As a result, cells can initiate anaphase with these merotelic kinetochores appearing to “lag” behind the segregating clusters of poleward moving kinetochores (Chunduri and Storchová, 2019; Cimini et al., 2004). It is these lagging chromosomes that are at high risk of forming micronuclei and suffering chromothripsis as the nuclear envelope reassembles (Zhang et al., 2015).

Why do merotelic attachments form? In prometaphase, as kinetochores undergo search-and-capture, there is a probability of binding microtubules emanating from opposite poles (Mitchison and Kirschner, 1985). The spindle geometry at nuclear envelope breakdown has been shown to affect this probability with reduced distance between spindle poles increasing the fraction of improper attachments (Kaseda et al., 2012; Marco et al., 2013; Silkworth et al., 2012). There is also evidence that the rate of microtubule-kinetochore turnover is important with a balance between having the necessary stability to enable chromosome movement, and sufficient turnover to limit the lifetime of improper attachments (Bakhoum et al., 2009a, 2009b). The turnover rate is cell-type specific and provides one explanation for increased chromosomal instability in cancer cells (Bakhoum et al., 2009a, 2009b). Physical properties of the kinetochore and the chromosome arms are also important, with increasing size elevating the risk of merotely and mis-segregation, respectively (Drpic et al., 2018; Worrall et al., 2018). Importantly, errors are not limited to merotely. For example, prolonged metaphase delay can lead to premature sister chromatid separation (PSCS) due to cohesion fatigue (Daum et al., 2011; Lara-Gonzalez and Taylor, 2012), and non-resolved syntelic attachments have been proposed as a source of non-disjunction (Cimini et al., 1999; Thompson and Compton, 2008; Torosantucci et al., 2009). However, the systematic detection of these different error events in pre-anaphase cells and establishing the causal relationships with segregation behavior in subsequent anaphase remains unresolved.

The major error correction mechanism in pre-anaphase cells is mediated by the Aurora B kinase, a component of the chromosome passenger complex (CPC), which localizes to the centromere-kinetochore interface (Broad et al., 2020; Lampson and Cheeseman, 2011). If Aurora B activity is compromised, the frequency of syntelic and merotelic attachments increase due to the failure to correct improper attachments (Cimini et al., 2006; Ditchfield et al., 2003; Hauf et al., 2003; Lampson et al., 2004). Aurora B is preferentially enriched on improper kinetochore attachments (Knowlton et al., 2006) where it phosphorylates outer kinetochore proteins, to destabilize attachments to microtubules (DeLuca et al., 2006; Welburn et al., 2010). As sister kinetochores form amphitelic attachments, increased tension and intersister distance have been proposed to reverse these destabilizing phosphorylations (Wang et al., 2011) and ultimately lead to microtubule attachment stabilization, which gradually increases during metaphase (Conti et al., 2019; Dunsch et al., 2011; Manning et al., 2010; Schmidt et al., 2010; Zhai et al., 1995). At the metaphase to anaphase transition, the motor protein MKLP2 relocates Aurora B to the spindle midzone (Gruneberg et al., 2004), where it generates a phosphorylation gradient (Fuller et al., 2008). This phospho-gradient has been suggested to delay chromosome decondensation and nuclear envelope reassembly (NER) in response to incomplete chromosome segregation during anaphase (Afonso et al., 2014).

The current paradigm in the field is that only rare kinetochores can escape pre-anaphase surveillance in non-transformed human cells resulting in a lagging chromosome rate of 5% (Bakhoum et al., 2009a, 2009b; Thompson and Compton, 2008; Worrall et al., 2018). This is five times higher than the risk of mis-segregation (see above). This discrepancy between the rate of lagging chromosomes and of mis-segregation suggests the existence of dedicated mechanisms to limit mis-segregation. However, how lagging chromosomes are defined is imprecise and often reliant on endpoint assays using fixed cell imaging, which do not detect chromosomes that lag after, or that are successfully segregated before, the fixation.

Here, we use a combination of lattice light-sheet imaging and computational analysis that enable a quantitative measure of chromosome lag to be defined. We term this “laziness” and reveal how a much larger proportion of kinetochores are at risk of mis-segregation than previously thought. By analyzing the history of lazy kinetochores, we identify key dynamic signatures in metaphase that predict the ultimate segregation outcome. Furthermore, we provide evidence that Aurora B operates during early anaphase to promote the rapid correction of the majority of lazy kinetochores. These data provide additional insight into the origins of chromosome mis-segregation and micronuclei formation in human cells.

## Results

### Lattice light-sheet imaging and automated analysis tools allow probing of the origins of chromosome segregation errors

**Figure 1:**
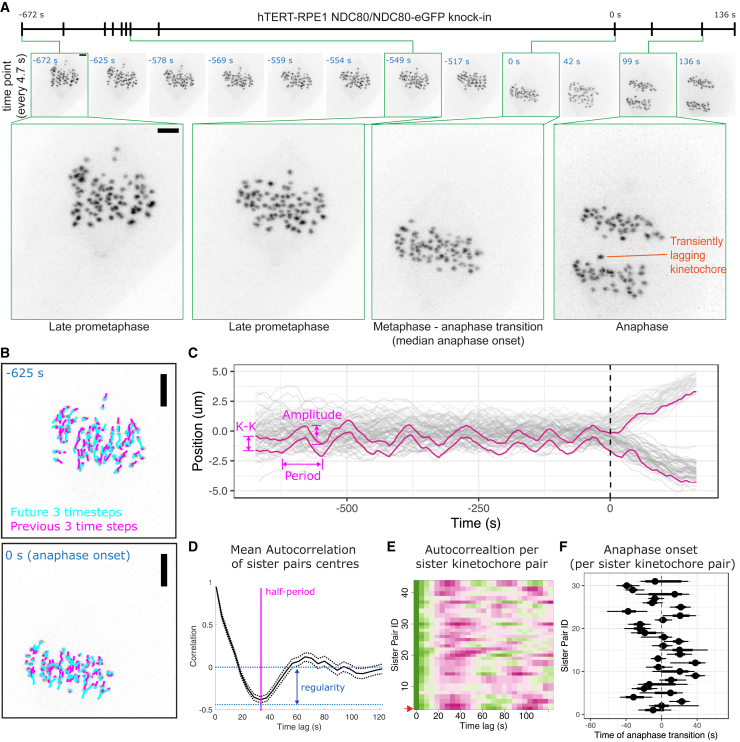
Lattice light-sheet microscopy enables imaging of mitosis with high spatiotemporal resolution and quantitative analysis of kinetochore dynamics (A) (Top panel) Z-projected movie stills of a cell progressing from late prometaphase to anaphase (time 0 = median anaphase onset time for this cell). Scale bar is 2 μm. (B) Example of kinetochore tracking using dragontails to annotate the previous and future 3 time steps in Z-projected movie stills. Scale bar is 3 μm. (C) Track overlay (x axis position in time, where the x direction is perpendicular to the metaphase plate along the spindle axis) shows trajectories of all sister kinetochore pairs (sister one in light gray; sister two in dark gray) during metaphase and anaphase. Magenta trajectories show the positions of a representative sister kinetochore pair that exhibit dynamic oscillations and timely segregation. Dashed line (time = 0) annotates the median anaphase onset time for this cell. Parameters related to kinetochore oscillations, intersister (K-K) distance, amplitude, and period, are shown. (D) Graph shows mean autocorrelation for all sister pair centers (kinetochore oscillations) in this cell. The time when the autocorrelation curve achieves its minimum corresponds to the half period of average kinetochore oscillations in this cell (35–40 s, annotated with pink line), and the depth at the minimum is the oscillation regularity (blue arrow). (E) Heatmap demonstrates the autocorrelation values (high values in green; low values in magenta) for each sister pair (each individual row) in this cell. Heatmap for the autocorrelation of the representative sister pair highlighted in (C) is Sister Pair ID = 1 (bottom row). (F) Median and 95% credible intervals are shown for the anaphase onset time of each sister pair from the cell in (A) (see mechanistic anaphase model in STAR Methods). Dashed line indicates the median anaphase onset time for this cell.

To understand the origins of chromosome segregation errors during mitosis, we used lattice light-sheet microscopy (Chen et al., 2014) to collect full 3D volumes every 4.7 s (s) for a total duration of between 3.1 and 17.2 min (min) (median 8.3 min). These image sequences capture events from late prometaphase through metaphase and anaphase onset to the late stages of anaphase (Figure 1A; Video S1). For this, we used a non-transformed near-diploid human hTERT-RPE1 cell line in which one allele of the NDC80 gene is tagged with eGFP (Roscioli et al., 2020). By adapting our existing kinetochore tracking (KiT) algorithms (Armond et al., 2016), we were able to capture an average of 34 ± 9 long tracks of paired sister kinetochores per cell, compared with the total diploid number (46 pairs), representing 74% ± 20%, (Figures 1B and S1). These long tracks lasted at least 75% of the duration of each movie providing a near-complete 3D view of kinetochore dynamics. All of the imaged cells entered anaphase, indicating an appropriate imaging setup for studying mitosis. Furthermore, population level analysis of kinetochore trajectories in metaphase confirms that sister kinetochore pairs underwent heterogeneous oscillatory motion with a half period of ∼40 s as previously described (Figures 1C–1E and S2A) (Armond et al., 2019; Dudka et al., 2019). This lattice light-sheet imaging and analysis pipeline thus captures dynamics of kinetochores at high temporal resolution from late prometaphase to late anaphase.

Video S1. 3D view movie of the cell shown in Figure 1, related to Figure 1A

Identifying the causal events involved in chromosome mis-segregation is hampered by the low rate of chromosome mis-segregation in hTERT-RPE1 cells (Worrall et al., 2018). To circumvent this, we used a standard nocodazole arrest-and-release procedure in order to increase improper attachments. We also confirmed that the nocodazole arrest-and-release had little impact on oscillation dynamics in metaphase (Figures S2A–S2F) suggesting kinetochore function is largely preserved.

**Figure 2:**
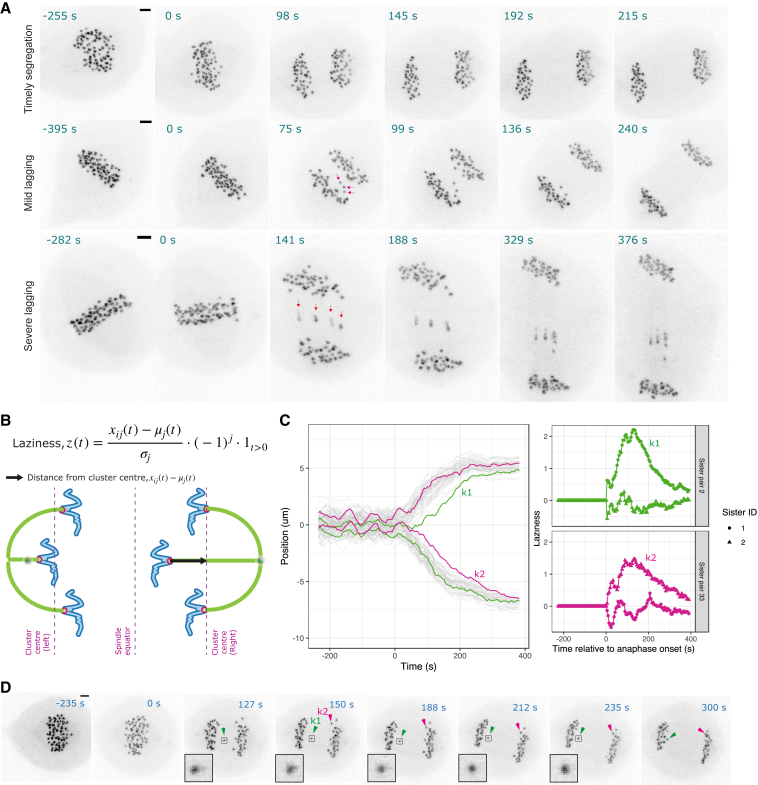
Laziness is a quantitative measure for spatiotemporal analysis of lagging chromosome behavior (A) Z-projected images of representative cells that undergo timely segregation or segregation with mild or persistent lagging chromosomes. (Middle panel) Magenta arrows annotate three kinetochores with different levels of lagging that later segregate correctly. (Bottom panel) Red arrows annotate multiple persistent lagging kinetochores that are mis-segregated. Scale bar, 2 μm. (B) Formula used for calculating laziness (z) of a kinetochore is illustrated on a cartoon (see STAR Methods). (C) (Left panel) Track overlay (x axis position in time) of a representative cell showing highlighted sister pair trajectories annotated for kinetochores k1 and K2. (Right panel) Laziness trajectory for k1 and K2 kinetochores and their sisters. (D) Z-projected images of the cell shown in (C). Scale bar, 2 μm.

To allow comparison of chromosome segregation behavior between cells, we developed an algorithm that segments trajectories into metaphase and anaphase. Estimates of anaphase onset times for the kinetochore pairs are shown in Figure 1F for the example cell displayed throughout Figure 1. The asynchrony in anaphase onset times between different sister pairs, as assessed by the median absolute deviation (a measure of spread similar to standard deviation but robust to outliers), was 16 ± 6 s for all cells (N=153 cells; DMSO, 2 h noc, 4 h noc pooled), consistent with earlier observations (Armond et al., 2019). Lagging chromosomes then manifest as a kinetochore that is delayed in segregating, or fails to segregate at all, compared with the two clusters of kinetochores that are moving toward opposite spindle poles (e.g., Figure 2A). Again, it is evident that defining kinetochores as “lagging” can be imprecise, i.e., when does late segregation become lagging? Indeed, we observed that kinetochores can exhibit a range of behaviors from timely segregation, and mild through to persistently lagging (Figure 2A). These various levels of “chromosome lag” may not be consistently identified in manual assessments by different experts in absence of a quantifiable definition.

### A quantitative measure for spatiotemporal analysis of lagging chromosome behavior

To study factors that affect the fidelity of chromosome segregation systematically and without bias, we developed an automated tool to identify lagging chromosomes from time series data. We assigned a quantitative measure, termed “laziness,” to reflect the individual segregation behavior of a kinetochore throughout anaphase. This allows us to analyze individual trajectories of kinetochores, and in this way reveal the time evolution of laziness during anaphase. We define the laziness, *z*, for an individual kinetochore at any given time point, based on its distance from the center of the cluster of kinetochores to which it belongs, a cluster comprises the kinetochores destined for one of the daughter cells (Figure 2B and STAR Methods). As a kinetochore’s lag increases behind its poleward moving cluster, the frame-to-frame laziness for that kinetochore takes increasingly high values (see the example lagging kinetochore k1 in Figure 2C and Video S2). This laziness over time is consistent with the movie image sequence of the same cell shown in Figure 2D. For most lagging kinetochores, laziness increases to a peak value, and subsequently reduces over time as the kinetochore returns to its segregating cluster later in anaphase (e.g., k1 in Figures 2C and 2D—see investigation of correction mechanisms below). On the other hand, kinetochores can trail behind their cluster with similar laziness dynamics except that the maximum value reached is lower (compare k2 with maximum (max.) laziness = 1.5 versus k1 with max. laziness = 2.2, in Figure 2C) and are not obviously lagging from the movie image sequence (k2 in Figure 2D). The laziness thus quantifies anaphase chromosome behavior and provides a firm basis for investigating the underlying mechanisms.

Video S2. Z-projected (maximum intensity) movie of the cell shown in Figure 2D, in which kinetochore k1 and its sister are annotated, related to Figure 2D

We next calculated the maximum laziness for each kinetochore throughout anaphase. This provides a readout that is independent of the duration and timing of a laziness event. The distribution of maximum laziness over the population of all kinetochore trajectories shows a sharp drop off and a long tail (n = 15,576; N = 153 cells; Figure S3A). As expected, we found a higher proportion of kinetochores with high maximum laziness after nocodazole arrest-and-release. While there is a clear spectrum of laziness, we investigated whether there is also a distinct population of kinetochores that exhibit high laziness. For this, we fitted the maximum laziness scores to a generalized extremal value distribution. Based on quantile-quantile (Q-Q) plots, we find that beyond a laziness threshold, *a*, of approximately 2, the quality of the fit breaks down (Figure S4B). This suggests that there is a distinct population of kinetochores that reach higher values of laziness during anaphase.

To further refine our estimate of this laziness threshold, *a*, and assess performance of the algorithm in identifying lagging kinetochores, we compared the output with (expert) manual inspection (Figure S4A). For this, we plotted a receiver operator characteristic (ROC) curve at various threshold settings (of *a*). The area under this curve (AUC) is a performance indicator that quantifies how good the performance of the algorithm is against manual inspection at distinguishing between cells with lagging kinetochores and those without. Random chance would achieve an AUC of 0.5, whereas using the maximum laziness gives an AUC of 0.88 (Figure S3B). Moreover, we can use the ROC curve to select a laziness threshold that gives us a false-positive rate (FPR, segregation delays detected by the algorithm, but not by the manual inspection; for example, Figure S3D) of only ∼5% (Figure S3C). This threshold (a=1.93) is close to the approximate value (∼2) derived from the extremal distribution (Figures S3A and S4B). With this threshold, we incur a false-negative rate of ∼30% (FNR, scored as lagging by manual assessment but not by the algorithm) although visual inspection showed that these events are largely missed due to mis-tracking as a result of low signal-to-noise ratio, or due to manual scoring based on kinetochore stretch (merotely) rather than solely on the distance of a kinetochore to the cluster (e.g., in Figure S3D). While this false-negative rate reduces the number of segregation errors scored by the algorithm, we prioritize minimizing false positives which could distort downstream analysis.

This threshold, *a*, allows kinetochores to be classified either as lazy, (maximum laziness >a) or timely (maximum laziness ≤a). Lazy kinetochores thus “lag” a significant distance behind their segregating cluster at some point during anaphase. This analysis reveals that the proportion of lazy kinetochores in DMSO-treated cells is 0.26%, which increases (3.3-fold) to 0.86% in nocodazole-treated cells in a duration dependent manner (Figure S3E). We also quantified the proportion of cells that contain at least one lazy kinetochore at any point during anaphase: 18% for DMSO-treated cells and increases to 44% with nocodazole treatment (Figure S3F). We note this is higher than the proportion of untreated cells with lagging chromosomes (5%–7%) found by fixed cell imaging in previous reports (Bakhoum et al., 2009a, 2009b; Thompson and Compton, 2008; Worrall et al., 2018). Thus, the number of chromosomes at risk of mis-segregation in human cells is considerably higher than previously thought.

### Lazy kinetochores have a distinct dynamic mitotic signature

**Figure 3:**
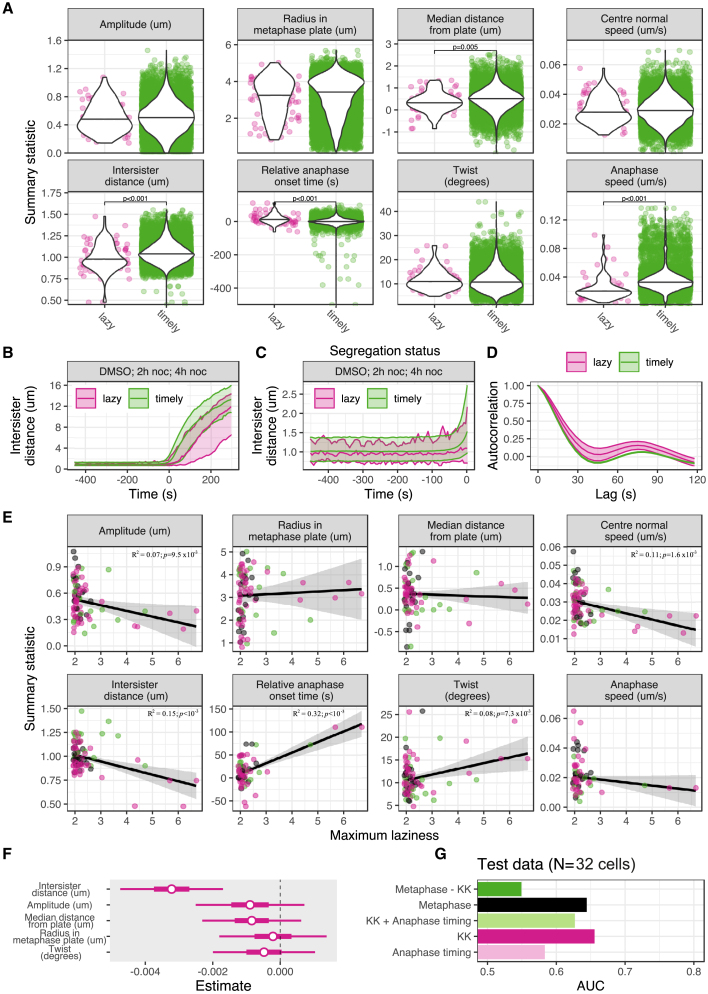
Lazy kinetochores have a distinct dynamic metaphase signature, which enables prediction of kinetochore fate in anaphase (A) Violin plots show medians of eight metaphase-anaphase variables (summary statistics) in lazy (with maximum laziness >1.93 threshold) and timely (with maximum laziness ≤1.93 threshold) kinetochores pooled from cells treated with DMSO, 2-h nocodazole or 4-h nocodazole. (B) Graph shows median intersister (K-K) distance (bands show 2.5%, 50%, and 97.5% quantiles) of all timely and lazy kinetochores throughout metaphase and anaphase. Time = 0 denotes anaphase onset. (C) Graph shows median intersister (K-K) distance throughout metaphase. (D) Graph shows average autocorrelation (standard errors with 0.95 confidence interval) for the metaphase oscillations of all timely and lazy kinetochores. Negative depth of the autocorrelation curve indicates the regularity of kinetochore oscillations. (E) Graphs show regression analyses of changes in the eight metaphase-anaphase variables (summary statistics) with respect to the maximum laziness throughout anaphase exhibited by lazy kinetochores pooled from cells treated with DMSO (gray, n=14 lazy kinetochores), 2-h noc (green, n=29 lazy kinetochores), or 4-h noc (magenta, n=48 lazy kinetochores). Black lines denote a linear fit to the data via maximum likelihood estimation, and the gray envelope shows the 95% confidence region for predictions from the model. *R*-squared and p values are shown for significantly correlating variables; for all variables see Table S1. (F) Plot shows estimated coefficients of metaphase variables in the predictive model (to estimate chromosome segregation in anaphase based on metaphase dynamics), as obtained by maximum likelihood estimation using pooled DMSO, 2-h- and 4-h-noc treated cells. Coefficients indicate the influence of each variable on the log-odds of whether a kinetochore is lazy. The influence of variables increases as the coefficient moves away from zero (dashed line). (G) Bar chart showing the area under the ROC curve (AUC) of several models using different covariates. The AUC captures the predictive capacity of each model in classifying lazy kinetochores based on metaphase dynamics. The models are trained on a pooled dataset (N=153 cells; DMSO [N=53], 2-h noc [N=46], 4-h noc [N=54]) and tested on a separate dataset of N=32 cells treated with DMSO. Covariates used in each model are as follows: metaphase, all metaphase variables shown in (F); KK, intersister (K-K) distance; anaphase timing, anaphase onset time of a sister pair relative to the median anaphase onset for the cell; metaphase − KK, all metaphase variables without K-K distance; anaphase timing + KK, relative time of anaphase onset and K-K distance.

Quantitative analysis of lattice light-sheet imaging provides 3D trajectory data going back in time to late prometaphase/early metaphase. This opens up the possibility of identifying whether there are any dynamic signatures in pre-anaphase kinetochores that can explain why certain kinetochores subsequently lag during anaphase. To do this, we separated kinetochores into those that underwent timely segregation (with maximum laziness ≤1.93 threshold) and those that exhibited segregation errors (with maximum laziness >1.93 threshold), henceforth referred to as lazy kinetochores. The kinetochore trajectory segments corresponding to metaphase were then extracted and analyzed. During metaphase, sister kinetochores undergo quasi-periodic oscillations along the spindle axis (Figures S2A and S2B) with the distance between the two sisters also breathing (Figures S2C and S2D) as kinetochores come under varying pulling and pushing forces (Burroughs et al., 2015; Jaqaman et al., 2010; Wan et al., 2012). We found that lazy kinetochores display reduced intersister (K-K) distance during metaphase, which persists through the metaphase-anaphase transition (Figures 3A–3C). Moreover, the lazy kinetochore population also initiated anaphase (sister separation) 14 s later (relative to the average time for kinetochore pairs in that cell), was located on average closer to the metaphase plate, and moved poleward with a lower speed in anaphase. In contrast, there was no significant difference between timely and lazy kinetochore populations in the oscillation amplitude, speed, position in the metaphase plate (radius), or twist (angle between inter-kinetochore axis and normal to metaphase plate) (Figure 3A). However, oscillations of lazy kinetochores were perturbed with a reduction in the regularity (Figure 3D, compare Figure 1D).

We reasoned that the intersister (K-K) distance may also scale with the severity of laziness. We found that among lazy kinetochores the reduction in K-K distance does indeed correlate with an increasing maximum laziness ($\text{p}<10^{-3}$, Figure 3E). Furthermore, a decreasing oscillation amplitude and speed and an increasing anaphase onset delay and twist also significantly correlate with increasing maximum laziness (Figure 3E). Oscillation amplitude and speed are not significantly different when comparing the medians of timely versus lazy kinetochores (Figure 3A), which likely reflects the considerable heterogeneity in oscillatory dynamics (Figure 1E). However, when restricted to only the lazy kinetochore population, correlations with maximum laziness are significant for these parameters (Figure 3E), suggesting that increased laziness of a kinetochore is associated with impaired metaphase oscillations. These trends are consistent with the small number of lazy kinetochores (n=22) in DMSO-treated cells and therefore do not reflect any issues associated with the nocodazole treatment (Figure S5A). Crucially, these data identify a dynamic metaphase signature that is associated with subsequent segregation fate during anaphase.

### Predicting kinetochore fate in anaphase based on its metaphase dynamics

Can this metaphase signature be used to successfully forecast whether a kinetochore will be lazy in the subsequent anaphase? To address this question, we fitted a logistic regression model to determine which variables are most influential in predicting whether a kinetochore will be timely or lazy in anaphase (see STAR Methods). Among covariates describing metaphase, the intersister (K-K) distance was the most influential variable because its model coefficient has the largest magnitude, and the 95% confidence interval [−0.040−0.014] does not contain zero. Confidence intervals for other variables indicate weaker evidence for the amplitude, twist, and the distance from the metaphase plate for an influence on the prediction (Figure 3F). We trained this simple predictive model on the pooled dataset of cells treated with DMSO, 2 h noc or 4 h noc (N=153 cells) and tested it on a separate set of DMSO-treated cells (N=32), examining the predictive potential of single and multiple variables (Figures 3G, S5B, and S5C). The AUC, the performance indicator, is 0.65 for the full model (using all metaphase variables shown in Figure 3F) on the test data (Figure 3G), suggesting that the predictive model clearly outperforms random chance (0.50). Furthermore, K-K distance alone performs to a similar extent to the full model and is a more powerful predictor than the timing of anaphase onset on the test data (Figure 3G). Metaphase dynamics of a kinetochore therefore enables forecasting of whether it will be a timely or lazy kinetochore during the subsequent anaphase.

### Initial evidence for an anaphase error correction process

**Figure 4:**
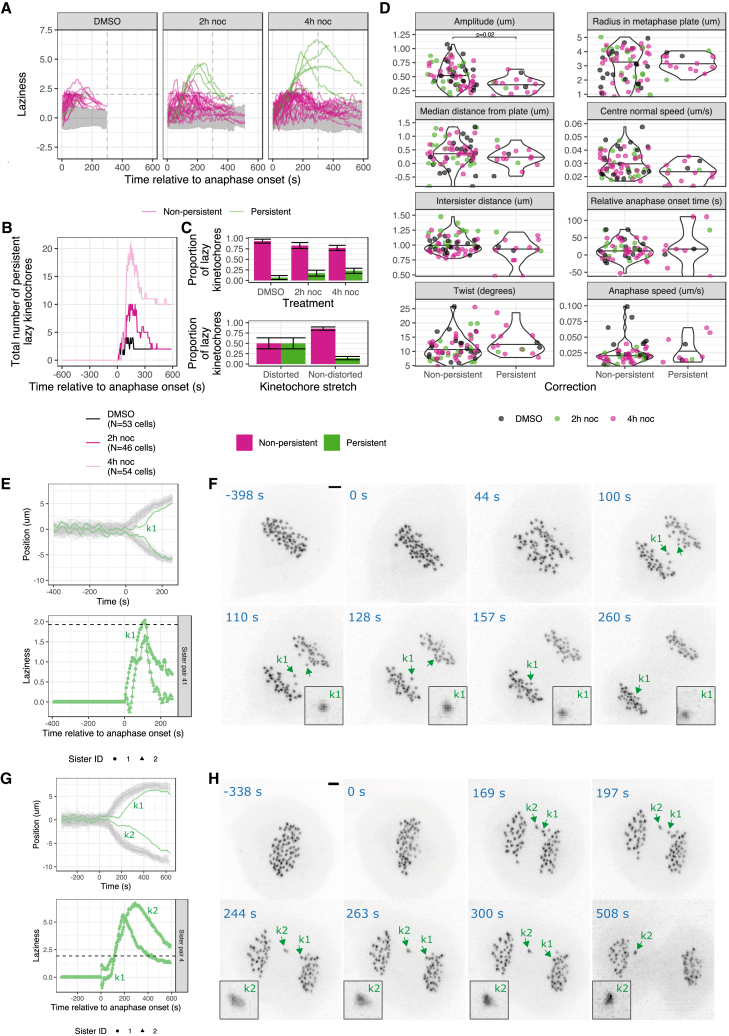
Correction of lazy chromosomes in anaphase is associated with dynamic kinetochore oscillations in metaphase (A) Graphs show laziness trajectories of lazy kinetochores (with maximum laziness >1.93 threshold) plotted over time. Black dashed line denotes laziness threshold (a=1.93); gray dashed line indicates 300 s after anaphase onset (end of anaphase A). Gray area indicates 2.5% and 97.5% quantiles of laziness corresponding to trajectories of timely kinetochores (with maximum laziness ≤1.93 threshold). Magenta trajectories are the lazy kinetochores that are corrected (laziness decreased below ≤1.93 threshold) within 300 s of anaphase onset. Green trajectories are the lazy kinetochores that persisted (laziness not decreased below ≤1.93 threshold). (B) Graph shows total number of persistent lazy kinetochores remaining with laziness >1.93 threshold throughout anaphase. (C) (Top panel) bar chart shows the proportions of corrected and uncorrected lazy kinetochores from cells treated with DMSO (N=53 cells), 2-h noc (N=46 cells) or 4-h noc (N=54 cells). (Bottom) Graph shows the proportions of corrected and uncorrected lazy kinetochores (n=91; pooled from cells treated with DMSO, 2-h noc or 4-h noc) with stretched kinetochores (n=14; 50% corrected) or unstretched kinetochores (n=77; 86% corrected) during anaphase. Fisher’s exact test indicates a significant difference, p=0.006. (D) Violin plots show eight metaphase-anaphase variables (summary statistics) in lazy kinetochores pooled from cells treated with DMSO, 2-h noc or 4-h noc and classified as corrected or uncorrected. Average values are median. (E) (Top) Track overlay shows the trajectory of a transiently lazy kinetochore k1 (and its sister) that is corrected within 300 s of anaphase onset. (Bottom) Graph shows the laziness trajectory of k1 kinetochore (and its sister) throughout anaphase. (F) Z-projected images of the cell in (E), where kinetochore k1 and its sister are annotated with green arrows. Zoomed images show that k1 is not distorted (unstretched) during anaphase. Scale bar, 2 μm. (G) (Top) Track overlay shows the trajectory of a lazy kinetochore K2 (and its sister) that is not corrected. (Bottom) Graph shows the laziness trajectory of K2 kinetochore (and its sister) throughout anaphase. (H) Z-projected image of the cell in (G), where kinetochore K2 and its sister (K1) are annotated with green arrows. Zoomed images show that K2 is distorted (stretched) during anaphase. Scale bar, 2 μm.

Plotting the laziness for individual kinetochores over time shows how most of the kinetochores segregate in a timely fashion (Figure 4A; gray areas correspond to timely kinetochores). Moreover, a majority of the lazy kinetochores are seen to reduce their laziness over time (magenta trajectories), while high laziness persists for a minority of kinetochores (green trajectories). To quantify this behavior, we calculated whether the frame-to-frame laziness, *z*, of a kinetochore reduced below the laziness threshold, a=1.93, within 300 s of anaphase onset—approximately the timescale for the end of anaphase A (Su et al., 2016; Vukušić et al., 2019) (henceforth referred to as early anaphase). After an initial increase in the number of lazy kinetochores following anaphase onset, the number of persistent lazy kinetochores decreases over time (Figure 4B). In DMSO-treated cells, 93% of lazy kinetochores exhibit transient lazy behavior and then correctly segregate (Figure 4C, top panel, and see example k1 in Figures 4E and 4F). However, a small proportion of lazy kinetochores (7%) persist beyond this time window (for example, see k1 and k2 in Figures 4G and 4H). In nocodazole-treated cells, the proportion of persistent lazy kinetochores is 3.3 times higher (23% in 4-h noc) and increases in a treatment duration dependent manner (Figure 4C, top panel).

One possibility is that the persistent lazy population simply reflects a slow, but normally attached, segregating kinetochore. On the other hand, they may potentially be the result of (a spectrum of) improper microtubule-kinetochore attachments (such as merotely) that were established pre-anaphase and then resolved in anaphase. We first compared the metaphase dynamics of lazy kinetochores that were either normally segregated or persistent. We found that the persistent lazy kinetochores have a reduced amplitude of oscillation (25% average reduction, from 0.5 to 0.38 μm) in metaphase (Figure 4D). Other variables do not show clear differences between the populations. Examples of a lazy kinetochore with high-amplitude oscillations that is subsequently segregated and a lazy kinetochore with impaired oscillations that persists are displayed in Figures 4E and 4F (Video S3) and Figures 4G and 4H (Video S4), respectively. This suggests that while a low K-K distance can predict subsequent lazy segregation (Figures 3A–3C), it is the kinetochores with dampened metaphase oscillatory dynamics (reduced amplitude) that are more likely to be persistently lazy in anaphase (Figure 4D).

Video S3. Z-projected (maximum intensity) movie of the cell shown in Figures 4E and 4F, in which kinetochore k1 and its sister are annotated, related to Figures 4E and 4F

Video S4. Z-projected (maximum intensity) movie of the cell shown in Figures 4G and 4H, in which kinetochore K2 and its sister are annotated, related to Figures 4G and 4H

If merotelic attachment is a contributor to the lazy behavior then we would expect some kinetochores to undergo distortion (stretching) as the correctly and incorrectly attached microtubules pull toward opposite poles (Cimini et al., 2004; Cojoc et al., 2016). Our high temporal resolution imaging enables us to capture dynamic kinetochore stretching and recoiling (reversal of stretching) events that manifest as the distortion of kinetochore spot shape. In fact, manual assessment of 3D movies showed that 14 of the lazy kinetochores (n=91) underwent distortion at varying degrees. Because of the opposing forces acting on merotelically attached kinetochores, we use spot distortion as evidence for kinetochore stretching due to merotely. Although microtubule density in anaphase makes attachments difficult to see, we could find an example of a stretched lazy kinetochore that was attached to both spindle poles (Figure S6). We next assessed whether the stretched lazy kinetochores differentiate from the unstretched lazy kinetochores in terms of their ability to be normally segregated. The stretched population (e.g., Figures 4G and 4H) of lazy kinetochores are more likely to persist in anaphase compared with the unstretched population (e.g., Figures 4E and 4F), 50% versus 14%, respectively (Figure 4C, bottom panel). These data show how merotely can be associated with lazy behavior but also hint at the presence of an “error correction” process in anaphase that can resolve such improper attachments.

### Aurora B inhibition disrupts the correction of chromosome segregation errors during anaphase

**Figure 5:**
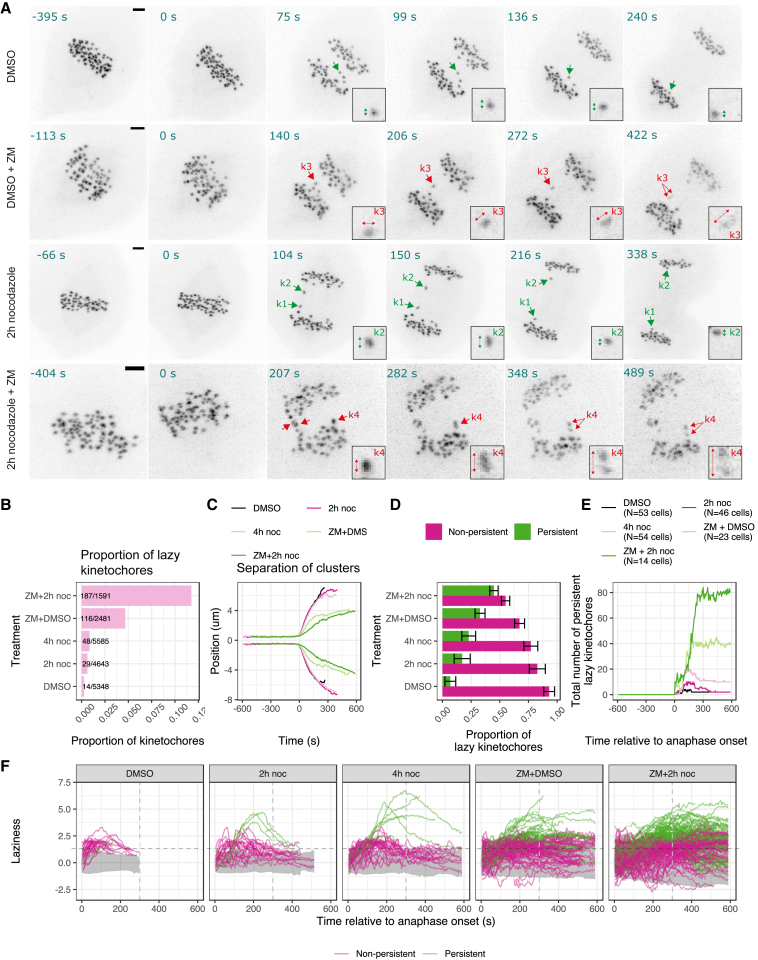
Aurora B kinase activity is required for correcting lazy kinetochores during anaphase (A) Z-projected images of a representative (top panel) cell that was treated with DMSO washout, which had multiple transient lazy kinetochores (green arrow) that are corrected in early anaphase; (second panel) cell that was treated with DMSO washout prior to being exposed to Aurora inhibitor (ZM447439), which had a lazy kinetochore k3 that was stretched and split into two parts, and was not corrected in anaphase; (third panel) cell that was treated with 2-h noc washout, which had lazy kinetochores k1 and k2, that were transiently stretched (and recoiled) and corrected in early anaphase; (bottom panel) cell that was treated with 2-h noc washout prior to being exposed to Aurora inhibitor (ZM447439), had multiple lazy kinetochores (k4 stretched and split into two parts), which were not corrected in anaphase. Scale bar, 2 μm. (B) Bar chart shows proportions (and numbers) of lazy kinetochores. (C) Track overlay shows the average position of segregating kinetochore clusters from all cells in a treatment group to compare the speed of kinetochore cluster segregation. (D) Bar chart shows the proportions of corrected and uncorrected lazy kinetochores from cells treated with DMSO (N=53 cells); 2-h noc (N=46 cells); 4-h noc (N = 54 cells); DMSO and then ZM (N=23 cells); 2-h noc and then ZM (N=14 cells). (E) Graph shows total number of uncorrected lazy kinetochores remaining with laziness >1.93 threshold throughout anaphase. Lazy kinetochores with laziness that fails to fall below the threshold, a=1.93, by the end of the movie are assumed to remain uncorrected. (F) Laziness trajectories of lazy kinetochores plotted over time. Black dashed line denotes laziness threshold (a=1.93); gray dashed line indicates 300 s after anaphase onset. Gray area indicates 2.5% and 97.5% quantiles of laziness corresponding to trajectories of timely kinetochores. Magenta trajectories annotate the lazy kinetochores that are corrected within 300 s of anaphase onset. Green trajectories annotate the lazy kinetochores that are persistent

Because Aurora B kinase activity is required for pre-anaphase error correction (Broad et al., 2020), we tested whether it is involved in the potential anaphase error correction process outlined above. To do this, we used the small molecule inhibitor, ZM447439 (ZM), of Aurora kinase (Ditchfield et al., 2003) following washout from nocodazole or DMSO. We only imaged the cells that were exposed to the Aurora inhibitor after their chromosomes had completed congression at the spindle equator; in other words, after they have “passed” Aurora-B-dependent error correction in prometaphase. Inhibition of Aurora B in this way still allowed anaphase to initiate but led to an increase in the proportion of lazy kinetochores (Figures 5A and 5B). Furthermore, Aurora B inhibition in fully aligned cells does not significantly affect metaphase kinetochore dynamics (Figure S7). We note that Aurora B inhibition does slow the overall separation of kinetochore clusters (Figure 5C), which is consistent with the anaphase roles of Aurora B reported previously (Hégarat et al., 2011; Uehara et al., 2013).

Loss of Aurora B activity dramatically increased the fraction of lazy kinetochores that persisted in anaphase (Figures 5D and 5E). For example, Figure 5A shows two lazy kinetochores (k3 and k4) that failed to be corrected, displaying severe spot distortion (kinetochore stretch). In fact, these kinetochores later disintegrated due to persistent pulling forces toward opposite poles. This is in contrast to cells with active Aurora B in which most lazy kinetochores are corrected during early anaphase (Figure 5D; see example kinetochores k1 and k2 in Figure 5A, which are corrected after transient kinetochore stretch). This effect was also observed in the cells that were treated with DMSO prior to Aurora B inhibition (see DMSO + ZM in Figure 5) demonstrating that the lack of anaphase error correction is not a consequence of nocodazole washout but establishes a broader paradigm relevant for untreated cells.

This failure to correct lazy kinetochores under Aurora B inhibition is also evident when plotting the laziness trajectories for individual kinetochores over time (Figures 5E and 5F). In cells with active Aurora B, even in those cases where laziness fails to fall below the threshold by 300 s (classified as persistent), the correction of lazy kinetochores appears to be in progress (green trajectories are declining, Figure 5F). In contrast, under Aurora inhibition (ZM), lazy kinetochores have a laziness that typically does not have a clear decline phase and remains high until the end of the movie (green trajectories show no obvious decline, Figure 5F). These differences, and the dynamics of early anaphase correction in cells with active Aurora B, are clearly visible in plots of the total number of persistent lazy kinetochores over time (Figure 5E).

**Figure 6:**
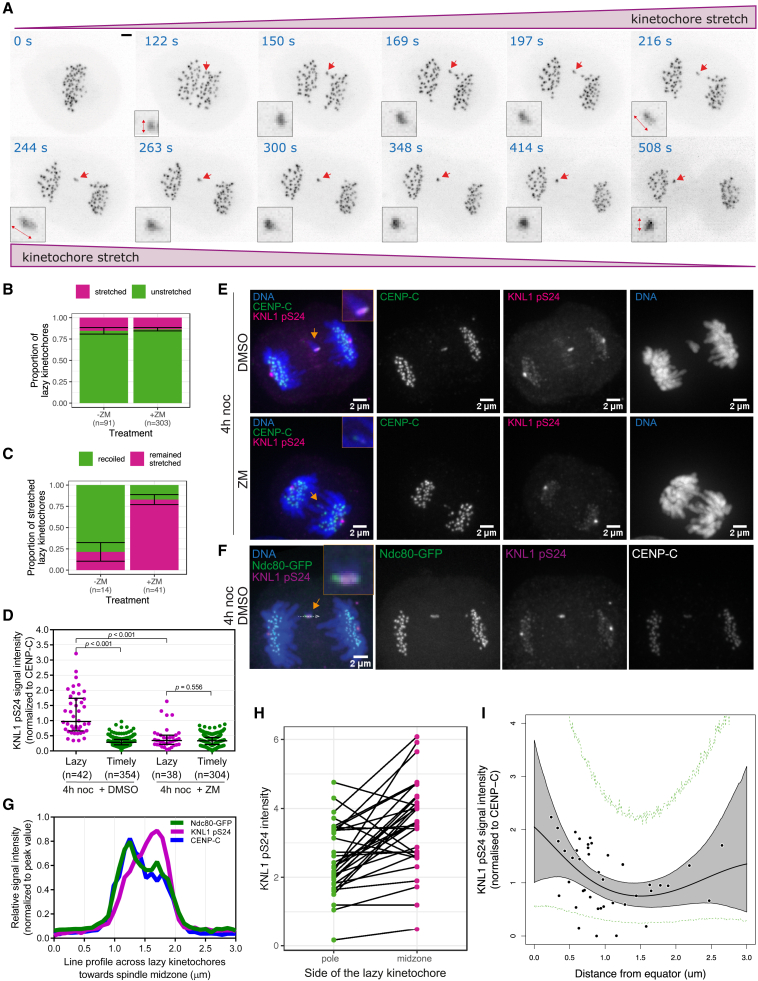
Aurora B generates a phosphorylation gradient that could drive error correction through attachment destabilization during anaphase (A) Z-projected images of a representative cell with active Aurora B highlighting stretch and recoil of a lazy kinetochore during anaphase. Red arrow annotates the stretched lazy kinetochore. Scale bar, 2 μm. (B) Bar chart showing the proportion of lazy kinetochores that undergo stretching with and without inhibition of Aurora B by ZM treatment. Fisher’s exact test indicates that there is no significant difference in the proportions between treatment groups. (C) Bar chart showing the proportion of stretched lazy kinetochores that undergo recoil or that remain stretched with and without inhibition of Aurora B by ZM treatment. Fisher’s exact test indicates a significant difference in the proportions between treatment groups, $\text{p}<10^{-3}$. (D) Graph shows Aurora-B-mediated KNL1 pS24 signal intensity on lazy and timely kinetochores from cells treated with 4-h noc arrest and release, followed by DMSO or Aurora B inhibitor (+ZM). p values report Mann-Whitney U test. Error bars indicate median ± SD. (E) Z-projected images from representative cells treated with DMSO or ZM for 10 min, following 4-h noc arrest—40 min washout, and prior to fixation. Fixed cells were stained with antibodies detecting CENP-C, KNL1 pS24, and with DAPI detecting DNA. Orange arrows annotate stretched lazy kinetochores. Scale bar, 2 μm. (F) Z-projected images from a representative cell treated with DMSO following 4-h noc arrest-and-release. Orange arrow indicates a stretched lazy kinetochore. Gray dashed arrow indicates a representative 3-μm line drawn through lazy kinetochores toward spindle midzone. Scale bar, 2 μm. (G) Graph shows average (median; n=32 lazy kinetochores) relative intensities (normalized to peak value on each kinetochore) of Ndc80-GFP, KNL1 pS24, and CENP-C signals over the 3-μm line profile shown in (F). (H) Relative intensity of KNL1 pS24 signal on the spindle-pole-facing side and midzone-facing side of a lazy kinetochore linked by black lines (n=32 lazy kinetochores). See changepoint model in STAR Methods. The relative intensity is significantly higher on the midzone-facing side compared with the poleward facing side (paired two sample Wilcoxon test, $p<10^{-3}$). (I) Graph shows how the Aurora B phospshogradient varies spatially. Based on measurements of the KNL1 pS24 signal intensity on lazy kinetochores (n=42, black points) in cells treated with DMSO (following 4-h noc), the black line is inferred showing the estimated gradient. The gray shaded region shows the possible variation in this gradient (95% credible region). Dashed green lines show 95% credible region for the data accounting for observation noise. See STAR Methods for details.

If Aurora B mediates this anaphase error correction by destabilizing erroneous microtubule-kinetochore attachments (as in pre-anaphase cells; Lampson and Cheeseman, 2011), we would predict that the fraction of stretched kinetochores (due to merotelic attachment) that remain stretched should increase upon Aurora inhibition. Indeed, this is what we observe. Figure 6A shows an example of a lazy kinetochore, from a cell with active Aurora B, that stretched and recoiled; whereas, Figure 5A (second and fourth rows) shows examples of stretched kinetochores that remained stretched in the absence of Aurora B activity. Quantification of these data show that following treatment with ZM, the proportion of lazy kinetochores that undergo stretching (consistent with merotelic attachment) does not change (Figure 6B), indicating that anaphase spindle forces are still sufficient to stretch merotelic kinetochores under Aurora inhibition. However, 83% of these stretched kinetochores remain stretched in ZM-treated cells compared with 21% in the cells with active Aurora B (Figure 6C). These data reveal that Aurora B activity is able to resolve merotely induced kinetochore distortion and promote the normal segregation of lazy kinetochores in anaphase.

### Aurora B generates a phosphorylation gradient that could drive error correction through attachment destabilization during anaphase

Our data predict that lazy anaphase kinetochores should be phosphorylated by Aurora B. We therefore imaged fixed anaphase cells that were stained with a phospho-specific KNL1 p-Serine24 antibody—an established marker of Aurora-B-dependent destabilization of kinetochore-microtubule interactions (Bajaj et al., 2018; Welburn et al., 2010). At anaphase, lazy kinetochores are clearly more phosphorylated at KNL1-Serine24 compared with timely segregating kinetochores (Figure 6D), and this phosphorylation is dependent on active Aurora B kinase (Figures 6D–6F). Moreover, we observed that the phosphorylation of lazy kinetochores is not uniform across the kinetochore (Figures 6E and 6F). Specifically, the average spatial distribution of KNL1 pS24 phosphorylation across lazy kinetochores relative to the kinetochore markers (CENP-C and Ndc80-GFP) shows preferential phosphorylation on the midzone-facing side (Figure 6G). Similarly, individual lazy kinetochores have significantly elevated phosphorylation on their midzone-facing side compared with the pole-facing side (Figure 6H) (see STAR Methods). These spatial differences in phosphorylation can be explained by the Aurora-B-dependent phosphorylation gradient (Afonso et al., 2014; Fuller et al., 2008). Reconstruction of the spatial phosphorylation gradient by Gaussian process regression (Rasmussen and Williams, 2006) from the KNL1 pS24 signal on multiple lazy kinetochores in anaphase (see STAR Methods) clearly illustrates a decay away from the midzone on a scale of around 1 μm (Figure 6I). These data indicate that midzone located Aurora B mediates attachment destabilizing phosphorylation of kinetochores in a gradient pattern.

### Impact of Aurora-B-gradient-mediated anaphase correction mechanism on chromosome segregation outcome

**Figure 7:**
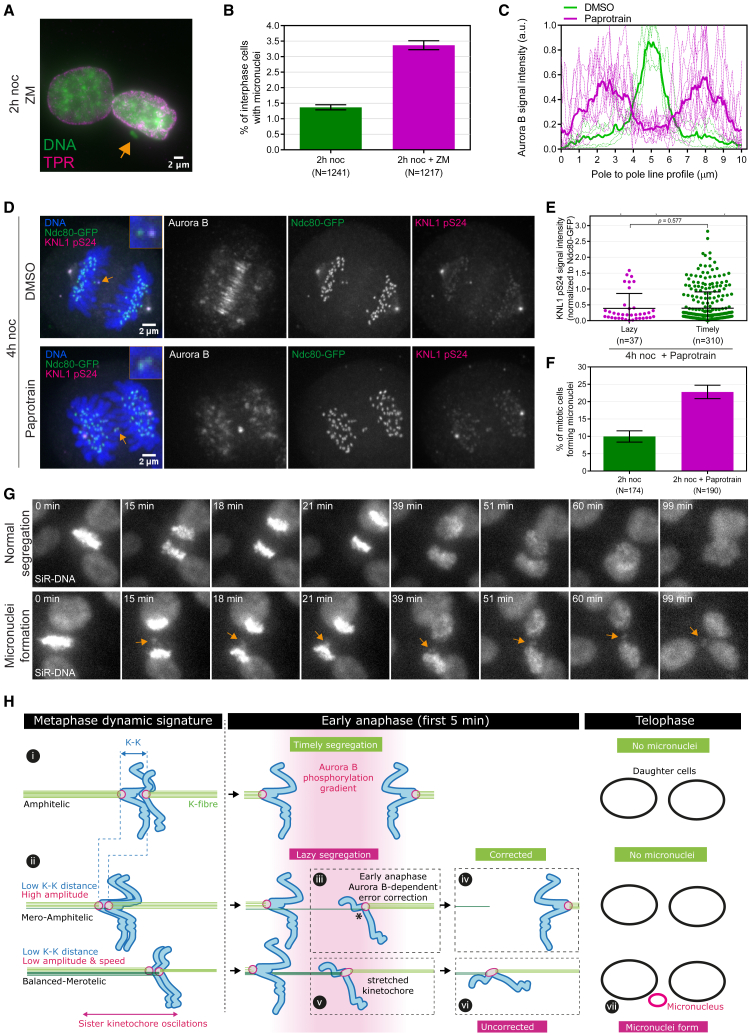
Aurora B gradient at spindle midzone corrects segregation errors that otherwise could form micronuclei (A) Z-projected image from a representative cell treated with Aurora inhibitor (+ZM) for 60 min, following 2-h noc arrest—40 min washout, and prior to fixation. Fixed cells were stained with the antibody detecting TPR, marking nuclear envelope, and with DAPI. Orange arrow annotates a cell with micronuclei formation. Scale bar, 2 μm. (B) Graph shows percentage of interphase cells with micronuclei. Error bars indicate mean ± SD. Mean values are average of results from two independent experiments (2-h noc, N = 631, N = 610; 2-h noc + ZM, N = 612, N = 605). (C) Graph shows Aurora B signal intensity over a 10-μm line drawn between spindle poles in each cell treated with 4-h noc washout followed by DMSO (N = 5 cells) or 10-μM paprotrain (N = 10 cells). Bold lines (green and magenta) denote the mean value for each treatment group. (D) Z-projected images from representative cells treated with DMSO or paprotrain for 35 min, following 4-h noc arrest—10 min washout, and prior to fixation. Orange arrows annotate stretched lazy kinetochores. Scale bar is 2 μm. (E) Graph shows KNL1 pS24 signal intensity on lazy and timely kinetochores from cells treated with 4-h noc arrest and release, followed by paprotrain. p value reports Mann-Whitney U test. Error bars show median ± SD. (F) Graph shows percentage of mitotic cells that formed micronuclei in the subsequent telophase. Error bars indicate mean ± SD. Mean values are average of results from two independent experiments (2-h noc, N = 102, N = 72; 2-h noc +paprotrain, N = 120, N = 70). (G) Z-projected images from representative mitotic cells with micronuclei formation (annotated with orange arrow) or normal segregation (without micronuclei). Cells were treated with DMSO or paprotrain following 2-h noc arrest and release and imaged in the presence of drugs for additional 2 h. (H) Working model explaining how attachment status and metaphase dynamics of kinetochores can affect their segregation behavior in anaphase. In metaphase, most kinetochores have amphitelic attachments (step i). However, a considerable proportion of kinetochores can have merotelic configuration ranging in severity from mero-amphitelic (with fewer mis-attached microtubules), to balanced-merotelic (with near equal numbers of microtubules on the correct and incorrect sides of a kinetochore) (step ii). In anaphase, mero-amphitelic kinetochores can be rapidly corrected via the Aurora B phosphorylation gradient (step iii and iv); whereas balanced-merotelic kinetochores become distorted (stretched) and possibly persist to telophase (step v and vi). Uncorrected lazy kinetochores may subsequently form micronuclei (step vii).

We next tested how loss of the anaphase error correction mechanism impacted the final outcome of mitosis. To do this, we quantified micronuclei formation rates by staining fixed cells with an antibody for a nuclear envelope marker (TPR) and with DAPI (DNA) (Figure 7A). Cells treated with ZM in anaphase (40 min after nocodazole washout) were more likely to form micronuclei (Figure 7B). This indicates a direct link between the reduced ability to correct lazy chromosomes (see Figures 5E and 5F) and micronuclei formation. To substantiate this finding, we treated cells with paprotrain, an MKLP2 inhibitor (Tcherniuk et al., 2010), to inhibit the localization of active Aurora B to the spindle midzone during anaphase (Figures 7C and 7D). Paprotrain treatment abolished the preferential phosphorylation of lazy kinetochores by Aurora B (Figures 7D and 7E). This further confirms that it is the Aurora B kinase pool that relocates to the spindle midzone, which is required for phosphorylation (and error correction) of lazy kinetochores. Consistently, paprotrain treatment led to a significant increase in the proportion of mitotic cells forming micronuclei during the subsequent telophase (Figures 7F and 7G). Upon MKLP2 inhibition, we observed an elevation of uncorrected lagging chromosomes but did not observe any chromosome bridges. These data suggest that attachment destabilizing phosphorylation of lazy kinetochores by the Aurora B gradient at the spindle midzone is involved in the prevention of micronuclei formation.

## Discussion

The scoring of the “lagging chromosome” phenotype underpins hundreds of cell biology studies on the genes and processes that give rise to chromosome mis-segregation. The lagging chromosome event has also gained widespread use as a proxy for the occurrence of merotelic kinetochore-microtubule attachments. Despite this, there is no convention on what constitutes a lagging chromosome. Most studies rely on fixed cell imaging which can only provide a snapshot of anaphase and is therefore sensitive to the “moment” in anaphase and to unavoidable observer bias i.e., when does a chromosome become lagging? This is particularly imprecise when using chromosome labels, since the position of bulky chromosome arms do not always represent the position of kinetochores where microtubules attach to segregate them. Recent efforts to quantify lagging chromosomes from fixed cell analyses are an important step forward (Gama Braga et al., 2021). However, these approaches do not use a time series and therefore cannot capture the temporal and spatial evolution of short-lived events. Here, we determined dynamic behaviors of kinetochores as they progress through metaphase and anaphase. This dynamic approach enables an objective analysis of lagging kinetochores to be developed; specifically, we define laziness, a quantitative measure of segregation behavior defined throughout anaphase. Lagging kinetochores are identified by high laziness values. We term these “lazy kinetochores” to retain reference to the underlying measure used for their identification.

This analysis reveals that there is a substantial population of kinetochores that become lazy during anaphase, with 18% of DMSO-treated cells possessing at least one kinetochore that became lazy at some point during anaphase. These kinetochores would be scored lagging by eye if observed at the time of maximum laziness. This is considerably higher than the proportion of cells with lagging chromosomes (5%–7%) found in previous reports based on fixed cell imaging (Bakhoum et al., 2009a, 2009b; Thompson and Compton, 2008; Worrall et al., 2018). Moreover, our algorithm uses a laziness threshold, tuned to an FPR of ∼5% against manual assessment. This threshold gives a ∼30% false-negative rate which implies we are still underestimating the lazy kinetochore population. This is predominantly because our algorithm is not able to detect lazy kinetochores with low levels of Ndc80 binding (low signal-to-noise ratio) nor does it include some highly stretched (merotelic) kinetochores that are close to the cluster (insufficiently lazy). Remarkably, this suggests that improperly attached kinetochores are not a rare event but a common feature of a normal unperturbed mitosis.

Our data show that a lowered K-K distance (a proxy for tension across the centromeric chromatin) and reduced regularity of oscillations in metaphase are a signature for lazy behavior of kinetochores during anaphase. Moreover, the severity of laziness correlates with the decrease in K-K distance along with lower oscillation speed and amplitude. Crucially, our predictive model is able to forecast lazy kinetochores in a new dataset of unperturbed cells. Thus, our findings are not restricted to cells that are arrested and released from nocodazole (comprising the majority of the training data). This indicates that problems in chromosome segregation are already present in metaphase.

What does the metaphase signature represent at the molecular level? Our data do not support a role for precocious sister chromatid separation (PSCS) because this would be expected to increase K-K distances and is normally the result of longer duration mitotic arrests (Worrall et al., 2018). This also suggests that our nocodazole arrest-and-release procedure is unlikely to affect cohesion between sister chromatids. The most natural interpretation of our data is that the metaphase signature reflects dynamic behavior of merotelically attached kinetochores in metaphase. Three observations would support this: (1) merotelic attachment would be consistent with the reduction in the K-K distance of the signature because of the pulling forces from the incorrect attachment bringing the merotelic kinetochore closer to its sister during metaphase, (2) it is well established that nocodazole arrest and release increases the number of lagging chromosomes with merotelic attachments (Cimini et al., 2001), and (3) we showed lazy kinetochores in anaphase that underwent stretching, an established feature of merotely (Knowlton et al., 2006).

However, we observed that only 15% of lazy kinetochores were stretched. To explain this, we propose the following working model: during metaphase sister kinetochores are expected to bind up to 20 microtubules from opposite spindle poles (Figure 7H, step i). A considerable proportion of these kinetochores would, however, have a merotelic configuration with reduced K-K distance. The severity of merotely would range from one or two mis-attached microtubules on the incorrect side (mero-amphitelic), through to full occupancy with equivalent numbers on the incorrect and correct sides of a kinetochore (balanced-merotelic; Figure 7H, step ii) (Gregan et al., 2011). This view of merotely is thus compatible with a range of segregation behaviors from chromosomes stuck at the midzone to mild delays in segregation. It is tempting to speculate that the number of mis-attached microtubules correlates with laziness in anaphase. We found that the most severe laziness associates with disrupted metaphase oscillations, which would be consistent with these merotelic kinetochores having balanced attachment to the two spindle poles, restricting their movement. As the cell progresses into anaphase, kinetochores with few mis-attached microtubules (mero-amphitelic) would be rapidly corrected (Figure 7H, step iii to iv); while those with more severe merotely (balanced-merotelic) would be distorted (stretched) and possibly persist to telophase (Figure 7H, step v to vi), and thus more likely to result in micronuclei formation (Figure 7H, step vii). This would also be consistent with previous microtubule-poison-based experiments that led to a model in which reduced kinetochore-microtubule occupancy (in metaphase) is associated with reduced K-K distance and an increase in lagging chromosomes (Dudka et al., 2018). The idea was that with fewer microtubules on both correct and incorrect sides of a merotelic kinetochore, there is a lower force differential between the sides, which leads to an attachment status closer to balanced merotely, which reduces the chances for correction.

The merotelic nature of lazy kinetochores is consistent with our finding that ∼93% of lazy kinetochores are corrected in early anaphase in unperturbed RPE1 cells and that this process requires Aurora B activity. Lazy kinetochores persist when Aurora B is inhibited, which increases the rate of micronuclei formation, hence identifying lazy kinetochores as possessing a potential for erroneous segregation. This leaves 2.3% (2/85) of unperturbed RPE1 cells with an uncorrected lazy kinetochore (our study), which is consistent with the 2% of telophase RPE1 cells that contain a micronucleus (Orr et al., 2021). We have thus identified an additional layer of anaphase error correction that is consistent with independent experiments showing that inhibition of Aurora B after anaphase onset also leads to an increase in lagging chromosomes in multiple cell types including RPE1 (Orr et al., 2021). We propose that the midzone Aurora B gradient promotes phosphorylation of kinetochore substrates to destabilize attachments during anaphase. This model is supported by our observation that stretched kinetochores recoil and move to the pole as incorrect microtubule attachments are lost, and that this is dependent on Aurora B activity. Furthermore, we have demonstrated that lazy kinetochores are phosphorylated by Aurora B at a site linked to destabilization of kinetochore-microtubule interactions (Bajaj et al., 2018; Welburn et al., 2010) and with higher phosphorylation on the midzone-facing side of the kinetochore. For stretched lazy kinetochores, extending ∼0.5 μm in the poleward direction, this increase on the midzone-facing side of the kinetochore (57% higher on average) is consistent with the estimated phosphorylation gradient (53% higher phosphorylation at 0.5 μm from the midzone compared with 1.0 μm from the midzone).

Recent work also indicates that the Aurora B midzone gradient mediates phosphorylation of outer kinetochore proteins during anaphase (Papini et al., 2021) on sites that have been shown to destabilize erroneous kinetochore-microtubule attachments in pre-anaphase cells (Welburn et al., 2010). We propose that after separating from its sister at anaphase onset, the incorrect attachment side of a merotelic lazy kinetochore is more likely to be closer to the midzone (asterisk in Figure 7H, step iii), hence within the Aurora B phospho-gradient. Aurora B would therefore destabilize the microtubules on the incorrect side more efficiently (Figures 6D–6I). If both sides of a merotelic lazy kinetochore are within the midzone phospho-gradient, Aurora B may reduce the stability of all microtubule attachments on both sides equally and catalyze the error correction through a tug-of-war between correct and incorrect sides, leading to the ultimate removal of the incorrect attachment. Failure to correct lazy kinetochores is associated with an increased risk of aneuploidy as a result of chromosome mis-segregation and/or micronuclei formation (even when the lazy chromosome ultimately moves toward the correct daughter cell). In fact, inhibition of Aurora B midzone localization eliminates attachment destabilizing phosphorylation of lazy kinetochores and elevates micronuclei formation (Figures 7C–7G).

This model is consistent with our observation that stretched lazy kinetochores (likely balanced merotelics) are less likely to correct (50% correction), while unstretched lazy kinetochores (likely mero-amphitelics) are efficiently corrected (86% correction) in early anaphase. In this regard, the probability of correction is also forecast by the behavior of a lazy kinetochore in metaphase—most notably by high oscillation amplitude. We suggest that higher amplitudes reflect kinetochores with fewer mis-attached microtubules (mero-amphitelic), which is consistent with findings reported in (Cimini et al., 2004). These kinetochores would be more efficiently corrected by Aurora B (Figure 7H, step iii). We would also predict that mero-syntelic attachments (with fewer attachments to the correct pole than to the incorrect pole) would “correct,” but result in non-disjunction due to earlier removal of the thinner correct attachment (Torosantucci et al., 2009). An important next step will be to establish dynamic signatures for these merotelic attachment variations, and to track their origins and fate during mitosis.

Our data provide evidence for Aurora-B-mediated destabilization of improper kinetochore-microtubule attachments in anaphase. However, we are not ruling out a possible contribution of anaphase spindle forces in the segregation of merotelically attached chromosomes to the correct daughter cell, potentially without the need for detachment (Cimini et al., 2004). Recently Orr et al. (2021) showed how depletion of KMN network components or inhibition of anaphase Aurora B increased the rate of lagging chromosomes and micronuclei formation and thus proposed that stable microtubule-kinetochore attachments are involved in anaphase error correction. They also suggested that midzone Aurora B activity increases microtubule-kinetochore attachment stability. Future work will be needed to understand the relationship between the attachment stabilization proposed by Orr et al. (2021), kinetochore stabilization proposed by Papini et al. (2021), and the destabilizing error correction proposed here.

In conclusion, we have established a quantitative framework to define kinetochore segregation behaviors and have dissected the mechanisms that cause and correct lazy kinetochores in anaphase. This has revealed how kinetochore behavior in metaphase forecasts their future and that a high proportion of kinetochores are at risk of mis-segregation without correction mechanisms. We have defined an additional layer of error correction, which operates in anaphase to ensure timely chromosome segregation and thus prevent micronuclei formation. This work provides firm ground for further investigations into the origins of whole chromosome aneuploidies—a hallmark of tumorigenesis and reproductive failure in humans.

### Limitations of the study

Our study has a number of technical limitations. Dual color imaging to directly visualize Aurora-B-dependent microtubule detachment or visualizing the attachment state of kinetochores in early anaphase is not technically feasible at present. Complete tracking through to telophase to determine the life history of kinetochores that generate micronuclei was also not shown. We are currently unable to efficiently track severely stretched kinetochores (due to substantial spot distortion) and profile dynamics of kinetochore shape, stretch, and recoil. Conceptually, our study could be expanded. Although the dynamic metaphase signature has predictive power for identifying (future) kinetochore laziness, there remains unexplained variability. It is likely that the model’s predictive power can be improved by including further statistics, such as chromosome size and identity.

## STAR★Methods

### Key resources table

**Table:** 

REAGENT or RESOURCE	SOURCE	IDENTIFIER / ACCESS
**Antibodies**		

Guinea Pig polyclonal anti-CenpC	MBL	Cat# PD030; RRID: AB_10693556
Rabbit polyclonal anti-Knl1-pS24	Welburn et al., 2010 (a gift from Iain Cheeseman)	https://cheesemanlab.wi.mit.edu/
Rabbit polyclonal anti-TPR (Abcam, ab59679)	Abcam	Cat# ab59679; RRID: AB_945913
Mouse monoclonal anti-Aurora B	BD Biosciences	Cat# 611083; RRID: AB_398396
Rabbit polyclonal anti-alpha tubulin	Abcam	Cat# ab4074; RRID: AB_2288001
Goat anti-guinea pig AlexaFluor 647	Invitrogen	Cat#A21450; RRID: AB_2735091
Goat anti-mouse AlexaFluor 594	Invitrogen	Cat# A11032; RRID: AB_2534091
Goat anti-rabbit AlexaFluor 594	Invitrogen	Cat#A11037; RRID: AB_2534095

**Chemicals, peptides, and recombinant proteins**		

Nocodazole	Sigma-Aldrich	Cat#M1404
ZM447439	Sigma-Aldrich	Cat#189410
Paprotrain	Merck	Cat#512533
Vectashield (with DAPI)	Vector	Cat#H-1200-10
SiR-DNA	Spirochrome	Cat#sc007
DMSO	Sigma-Aldrich	Cat#D2438

**Deposited data**		

Kinetochore tracks	This paper	https://doi.org/10.5281/zenodo.5551168

**Experimental models: Cell lines**		

RPE1 Ndc80-EGFP (MC191)	Roscioli et al., 2020	N/A

**Software and algorithms**		

KiT v2.3	This paper	https://github.com/cmcb-warwick/KiT
lazychromosomes v0.1	This paper	https://github.com/shug3502/lazychromosomes
Slidebook 6	3i	https://www.intelligent-imaging.com/slidebook
ImageJ / Fiji	Open source	https://imagej.net/software/fiji/

### Resource availability

#### Lead contact

Further information and requests for resources and reagents should be directed to and will be fulfilled by the lead contact, Andrew McAinsh (A.D.McAinsh@warwick.ac.uk).

#### Materials availability

Reagents generated in this study will be made available on request, but we may require a payment and/or a completed Materials Transfer Agreement if there is potential for commercial application.

### Experimental model and subject details

#### Cell culture, drug treatment and generation of cell lines

Immortalized (hTERT) diploid human retinal pigment epithelial (RPE1) cell line (MC191), expressing endogenously tagged Ndc80-eGFP, was generated by CRISPR-Cas9 gene editing (Roscioli et al., 2020). hTERT-RPE1 cells were grown in DMEM/F-12 medium containing 10% fetal bovine serum (FBS), 2 mM L-glutamine, 100 U/ml penicillin and 100 mg/ml streptomycin (full growth medium); and were maintained at 37°C with 5% ${\text{CO}}_{2}$ in a humidified incubator. For nocodazole arrest-and-release experiments cells were treated with DMSO (1/50,000 v/v) or 330 nM nocodazole for 2h or 4h, followed by transferring the coverslip to 5 ml full growth medium without any drugs, and 20 min incubation (washout step). After the washout, the coverslip was transferred to the lattice light sheet microscope (LLSM) bath filled with ${\text{CO}}_{2}$-independent L15 medium, where live imaging takes place. For Aurora B kinase inhibitor (ZM447439) treatment cells were treated with DMSO (1/50,000 v/v) or 330 nM nocodazole for 2h, followed by transferring the coverslip to 5 ml full growth medium without any drugs, and 30 min incubation (washout step). After the washout, coverslip was transferred to the LLSM bath filled with ${\text{CO}}_{2}$-independent L15 medium, including 4 *μ*M ZM447439, where live imaging takes place.

### Method details

#### Live cell imaging by Lattice light sheet microscope (LLSM)

The lattice light sheet microscope (Chen et al., 2014) used in this study was manufactured by 3i (https://www.intelligent-imaging.com). Cells were seeded on 5 mm radius glass coverslips one day before imaging. On the imaging day, cells were treated with drugs, and the coverslip was transferred to the LLSM bath filled with ${\text{CO}}_{2}$-independent L15 medium, where live imaging takes place. All imaged cells entered anaphase, which is a suitable proxy for a lack of phototoxicity effects (Jaqaman et al., 2010). The LLSM light path was aligned at the beginning of every imaging session by performing beam alignment, dye alignment and bead alignment, followed by the acquisition of a bead image (at 488 nm channel) for measuring the experimental point spread function (PSF). This PSF image is later used for the deconvolution of images. 3D time-lapse images (movies) of Ndc80-eGFP were acquired at 488nm channel using 1% laser power, 50 ms exposure time/z-plane, 93 z-planes, 307 nm z-step, which results in 4.7 s/z-stack time(frame). Acquired movies were de-skewed and cropped in XYZ and time, using Slidebook software in order to reduce the file size. Cropped movies were then saved as OME-TIFF files in ImageJ.

#### Manual assessment of lazy kinetochores

Movies for each cell were manually assessed by searching for prominent lagging kinetochores using the 3D view mode of Slidebook software (to avoid projection effects in the *Z* direction). Cells that had rotated on the coverslip during image acquisition were reoriented such that their segregation axes would correspond to the direction perpendicular to the metaphase plate according to the observer. This ensures an optimal cell orientation for viewing the segregation dynamics, and thus was used for the manual assessment. A list of cells ranked by maximum laziness (detected in each cell) was compared (see Figure S3A) with the results of manual inspection of the same data (N=153 cells; DMSO, 2h noc, 4h noc pooled). This revealed that the algorithm identified kinetochores with high laziness (threshold = ∼2 based on extremal analysis) within the subpopulation of cells that had been manually recorded as having zero prominent lagging kinetochores. Manual reassessment of these cells revealed that 12 of them did indeed have lagging kinetochores which had not been noticed in the first manual inspection. Cells in the ranked list were then reassessed until it was clear no further lagging kinetochores were found. Any additional cells with lagging kinetochores were included in the population of cells classified as having manually assigned lagging kinetochores. This list was used to calibrate the algorithm’s laziness threshold.

#### Deconvolution and kinetochore tracking

Movies were deconvolved using the Richardson-Lucy algorithm for deconvolution via the Flowdec library (Czech et al., 2019). The PSF used was a non-isotropic 3D Gaussian PSF fitted to the measured experimental PSF from each imaging session. Gaussian noise similar to background was added to blank regions of the image to avoid artefacts at the boundaries to blank image regions. Kinetochore tracking (KiT v2.3) software (Armond et al., 2016) was used to detect and track kinetochores, and subsequently pair sister kinetochores. Detection is achieved via adaptive thresholding of movies, and refined via a Gaussian mixture model. Detected kinetochores are linked between frames to form tracks via a Kalman filter and linear assignment problem. Tracks are grouped based on metaphase dynamics via a linear assignment problem. A plane is fitted to the metaphase plate as a reference coordinate system, in which the *x* direction points perpendicular to the metaphase plate, and *y* and *z* lie within the plate.

Statistics on the number of pairs tracked per cell are shown in Figure S1, and the majority of kinetochore pairs are tracked for more than 75% of the movie (Figure S1A). To ensure that we do not miss tracked kinetochore pairs with short tracks that exhibit lazy behaviour, we assess laziness for these short kinetochore tracks and include these in our analysis. When these short tracks are included, a total of more than 92 kinetochore tracks per cell can result, since such a kinetochore that is tracked early in the movie, disappears and subsequently is tracked later in the movie can be counted twice.

#### Immunofluorescence microscopy

RPE1 cells, stably expressing Ndc80-EGFP, were fixed in 10 mM EGTA, 1 mM MgCl_2_, 20 mM PIPES pH 6.8, 0.2% Triton X-100, and 4% formaldehyde for 10 min, washed 3 times in PBS before incubation in PBS with 3% BSA for 30 min to block non-specific antibody binding. Next, cells were incubated with primary antibodies for 12 h at 4°C, washed 3 times in PBS and then incubated for 60 min with secondary antibodies (Alexa Fluor, ThermoFisher Scientific). All antibodies were diluted in PBS + 3% BSA. Cells were then washed 3 times in PBS and mounted on Vectasheild with DAPI. Primary antibodies: Anti-CENP-C pAb (Guinea Pig) (MBL, PD030); anti-KNL1 pSerine24 pAb (Rabbit) (A gift from Iain Cheeseman); anti-TPR pAb (Rabbit) (Abcam, ab59679); anti-Aurora B (mouse) mAb (BD Biosciences), anti-*α*-tubulin (Rabbit) (Abcam, ab4074). Image stacks were acquired on Marianas spinning disk confocal microscope (3i, Intelligent Imaging Innovations) equipped with 100X / 1.4 NA oil-immersion objective. Image stacks were acquired over 75 z-slices separated by 200 nm using the 405, 488, 561 and 640 nm wavelength lasers. Kinetochore signals were quantified on ImageJ by measuring signal intensities within identical ROIs. Kinetochore line profiles were obtained by measuring signal intensities over a 3 *μ*m line drawn towards spindle midzone through lazy kinetochores. For the assessment of micronuclei formation in fixed cells, image stacks were acquired on API Deltavision Elite microscope equipped with 20X air objective, over 7 z-slices separated by 2 *μ*m using the 405 and 561 nm wavelength lasers. Cells with micronuclei were scored by eye on ImageJ.

#### Live cell imaging for the assessment of micronuclei formation

RPE1 cells, stably expressing Ndc80-EGFP, were seeded on glass bottom FluoroDish (FD35-100, World Precision In-strument, Inc.) one day prior to imaging. Next day, cells were treated with 330 nM Nocodazole and 250 nM SiR-DNA (Spirochrome) for 2h, and then washed out with full growth media. 10 minutes after the washout, cells were treated with DMSO or 10 *μ*M Paprotrain (MKLP2 inhibitor). Time-lapse imaging (2-3h) was performed when mitotic cells started entering anaphase (∼35 min after washout), using Olympus DeltaVision microscope (Applied Precision, LLC) equipped with 40X objective and Photometrics CoolSNAP HQ (Roper Scientific) camera. Temperature (37°C) and CO_2_ levels (5%) were held constant. Image stacks were acquired over 7 z-slices separated by 2 *μ*m, every 3 min, using the 640 nm (Cy5 filter) wavelength laser. During each 3 min time/frame ∼25 cell fields (1024x1024 pixels) were visited. Micronuclei formation was scored by eye using ImageJ.

### Quantification and statistical analysis

#### Mechanistic anaphase model

A hierarchical model was used to describe the positions of each kinetochore pair in a cell. The model takes the form of a stochastic differential equation with terms for the spring force due to chromatin connecting sister kinetochores, the polar ejection force, and forces due to microtubule polymerization/depolymerization, as in previous work (Armond et al., 2015). In metaphase, the following dynamics hold for the position, $X_{t}^{j}$, of sister kinetochore *j* at time *t*:

$$dX_{t}^{1}=\left(-v_{\sigma_{t}^{1}}-\kappa \left(X_{t}^{1}-X_{t}^{2}-L\;\cos\;\theta_{t}\right)-\alpha X_{t}^{1}\right)dt+sdW,$$

$$dX_{t}^{2}=\left(v_{\sigma_{t}^{2}}-\kappa \left(X_{t}^{2}-X_{t}^{1}+L\;\cos\;\theta_{t}\right)-\alpha X_{t}^{2}\right)dt+sdW,$$

which comprises the mechanical forces $v_{\sigma_{t}^{j}}$ due to K-fibre polymerisation/depolymerisation associated with the hidden K-fibre state $\sigma_{t}^{j}$ (polymerising (+), depolymerising (-)), a centromeric spring force between the sisters, $\kappa \left(X_{t}^{1}-X_{t}^{2}-L\;\cos\;\theta_{t}\right)$, spring constant *κ* and natural length *L*, projected to the *x*-axis (sister-sister twist $\theta_{t}$), the polar ejection forces with proportionality constant, *α*, and the thermal fluctuations with standard deviation *s*.

In anaphase, the polar ejection force and the chromatin spring force are assumed to be absent, giving:

$$dX_{t}^{1}=v_{A}dt+sdW,$$

$$dX_{t}^{2}=-v_{A}dt+sdW.$$

with anaphase speed $v_{A}$. We assume there are no effects of slowing down on kinetochore segregation over the timescale considered such that a single speed, $v_{A}$, can describe how kinetochores separate as they segregate. An additional anaphase reversal state, only accessible from the anaphase state, is included in the model to account for reversals in anaphase such that

$$dX_{t}^{1}=2sdW,$$

$$dX_{t}^{2}=2sdW.$$

where we assume kinetochores are diffusing.

The K-fibre polymerisation state is described by $\sigma_{t}^{j}\in \left\{+,-,A,R\right\}$, in metaphase, either polymerising (+) or depolymerising (-), and in anaphase, either moving towards their pole (*A*) or in a reversal reversal (*R*). This discrete hidden state evolves as a Markov process. Biophysical parameters are assumed individual to each kinetochore pair, while parameters governing the transitions between hidden states are assumed to be shared between all kinetochore pairs in a cell giving a hierarchical cell based model.

To fit the hierarchical anaphase model to experimental data, we take a Bayesian approach and draw samples from the posterior distribution via Markov chain Monte Carlo (MCMC). Specifically, we use the No-U-Turn-Sampler (NUTS) (Hoffman and Gelman, 2014) implementation of Hamiltonian Monte Carlo (Neal, 1994) in the Stan software platform (Carpenter et al., 2017). The likelihood is evaluated via the forward algorithm (Rabiner, 1989). Convergence of MCMC chains is assessed via the Gelman-Rubin $\hat{R}$ statistic (Gelman and Rubin, 1992; Vehtari et al., 2021) using only results where $\hat{R}<1.10$ for all parameters. Across all treatment groups, MCMC chains converged for 224/259 cells, and among these cells for 7040/7253 kinetochore pairs (Figures S1D and S1E) which were used in subsequent analysis.

The mechanistic anaphase model was fitted to long trajectories from each cell, annotating the trajectory by sister direction and anaphase separation time for each pair. Based on the estimates of anaphase onset times, we obtained an estimate of the median time of anaphase onset for a cell. Using this estimate of the median time of anaphase onset for a cell, we assessed the laziness for all tracked kinetochores (including kinetochore pairs with short tracks, and unpaired kinetochores).

#### Definition of the laziness, *z*

The laziness of a kinetochore at time, *t*, is given by

$$z\left(t\right)=\frac{x_{ij}\left(t\right)-\mu_{j}\left(t\right)}{\sigma_{j}}\cdot {\left(-1\right)}^{j}\cdot 1_{t>0},$$

where $x_{ij}\left(t\right)$ is the position of kinetochore *j* from sister pair *i* (measured relative to the metaphase plate), $\mu_{j}\left(t\right)$ is the median position of the daughter cell cluster *j*, and $\sigma_{j}$ is a scale for the spread of daughter cell cluster *j* estimated via the median absolute deviation on 20 early anaphase frames. The $\frac{x_{ij}\left(t\right)-\mu_{j}\left(t\right)}{\sigma_{j}}$ term is similar to the definition of a *z* score with reference to a normal distribution. The ${\left(-1\right)}^{j}$ term ensures that z(t) is positive for kinetochores between clusters, i.e. indicative of slower segregation than the median. The indicator term $1_{t>0}$ makes z(t) equal to 0 prior to anaphase onset (at t≤0). The median position of the daughter cell cluster is used for $\mu_{j}\left(t\right)$ to ensure robustness of the laziness statistic with respect to tracking errors. Software to compute laziness from kinetochore tracks is available at https://github.com/shug3502/lazychromosomes.

#### Summary statistics to describe dynamics of kinetochores in metaphase and anaphase

The intersister (K-K) distance (see Figure 1C) is calculated in 3D for a kinetochore pair as $d_{KK}=\sqrt{{\left(x_{i1}\left(t\right)-x_{i2}\left(t\right)\right)}^{2}+{\left(y_{i1}\left(t\right)-y_{i2}\left(t\right)\right)}^{2}+{\left(z_{i1}\left(t\right)-z_{i2}\left(t\right)\right)}^{2}}$ where $x_{ij}\left(t\right),y_{ij}\left(t\right),z_{ij}\left(t\right)$ are the position in each coordinate of kinetochore *j* from sister pair *i* at time *t*, with the *x* coordinate perpendicular to the metaphase plate. All metaphase summary statistics are calculated across a trajectory excluding the 60 s prior to anaphase onset, and summarised via the median. To calculate the amplitude of oscillation of an individual kinetochore (see Figure 1C), we used a sliding window of 20 frames and calculated the amplitude as $A=\left(\text{max}\left(x_{ij}\left(t\right)\right)-\text{min}\left(x_{ij}\left(t\right)\right)\right)/2.$ The average distance from the metaphase plate is calculated as $d_{MP}={\left(-1\right)}^{1+j}\text{median}\left(x_{ij}\left(t\right)\right)$ using a signed distance to indicate perpendicular distance from the metaphase plate in the direction of the cluster towards which the kinetochore will segregate. The radius within the metaphase plate is calculated as $r=\sqrt{y_{ij}{\left(t\right)}^{2}+z_{ij}{\left(t\right)}^{2}}.$ The centre normal speed of a kinetochore pair is calculated as the framewise speed of the mean position of the pair as follows $v_{CNS}=\left(x_{ij}\left(t+\text{Δ}t\right)+x_{ij}\left(t+\text{Δ}t\right)\right)-\left(x_{i1}\left(t\right)+x_{i2}\left(t\right)\right)/2\text{Δ}t$ where Δt is the time step between frames, and this is summarised via the standard deviation across a trajectory to capture the scale of changes in speed over an oscillation. The twist angle of a kinetochore pair is computed as ${\text{cos}}^{-1}\left(\text{median}\left(\left|\text{cos}\left(\phi \right)\right|\right)\right),$ where $\text{cos}\left(\phi \right)=\left(x_{i2}\left(t\right)-x_{i1}\left(t\right)\right)/\left||x_{i2}\left(t\right)-x_{i1}\left(t\right)|\right|$ with $x_{ij}\left(t\right)={\left(x_{ij}\left(t\right),y_{ij}\left(t\right),z_{ij}\left(t\right)\right)}^{T}$. The relative anaphase onset time onset is calculated as $t_{A}^{i}-\text{median}\left(t_{A}^{i}\right)$ where the median is calculated across kinetochore pairs, and $t_{A}^{i}$ is the median estimate of anaphase onset for a pair based on the mechanistic anaphase model. The anaphase speed, $v_{A}$, is a 1D speed in the direction perpendicular to the metaphase plate, as in the mechanistic anaphase model described above.

#### Logistic regression model

The logistic regression model is a generalized linear model to express the relationship between a binary dependent variable, *y*, (here corresponding to whether a given kinetochore has laziness above the threshold, a=1.93) and a matrix, X, of covariates (summary statistics describing dynamics of the given kinetochore in metaphase only). Suppose that p=P(kinetochore is lazy). We assume a linear relationship between the predictor variables and the log-odds, $\eta =\text{log}\left(\frac{p}{1-p}\right)$, of a kinetochore being lazy such that $\eta =b_{0}+b^{T}X$. We obtain

$$p=\frac{1}{1+\text{exp}\left[-\left(b_{0}+{\left(b\right)}^{T}X\right)\right]}.$$

We consider five models based on different combinations of covariates: 1) Metaphase covariates (K-K distance, amplitude, median distance from metaphase plate, radius in metaphase plate, twist); 2) K-K distance only; 3) time of anaphase onset relative to the median for a cell, only; 4) K-K distance and time of anaphase onset relative to the median for a cell; 5) Metaphase covariates without K-K distance (amplitude, median distance from metaphase plate, radius in metaphase plate, twist). Each model is fitted based on maximum likelihood estimation to data from N=153 cells (n=10160 kinetochores; DMSO, 2h noc, 4h noc pooled). Predictions are made for all kinetochores in N=32 DMSO cells unseen by the models to evaluate performance.

#### Changepoint model to quantify phosphorylation on each side of lazy kinetochores

To quantify how much KNL1 pS24 signal is on each side of a lazy kinetochore, we measured line profiles across each stretched lazy kinetochore from the poleward side towards the mid-zone facing side and fitted a changepoint model to identify two changepoints, $c_{1}$ and $c_{2}$, defining the ends of the kinetochore. The changepoint model assumes that for a kinetochore marker there is a background level of signal, *b*, away from the kinetochore and a different signal level, *a*, on the kinetochore itself. Thus, assuming a Gaussian observation error, we have for each pixel measured in the line profile:

$$y_{i}∼N\left(s,\sigma \right),$$

where $y_{i}$ is the signal in the kinetochore marker channel for pixel *i*, and

$$s=\left\{ \begin{array}{ll} b\;\text{ if }i<c_{1}, & \\ a\;\text{ if }c_{1}\leq i<c_{2}, & \\ b\;\text{ if }i\geq c_{2}. & \end{array}\right.$$

This model is fitted to the Ndc80 channel data using an MCMC method utilising an Hamiltonian Monte Carlo algorithm and implemented in Stan (Carpenter et al., 2017). The discrete latent states, $c_{1}$ and $c_{2}$, are marginalized out during inference.

We determine the centre of the kinetochore as $c=\left(c_{1}+c_{2}\right)/2$, and assess the KNL1 pS24 signal on the poleward side as the sum of all pixels in the line profile such that $c_{1}<i\leq c$, and similarly on the mid-zone facing side as the sum of all pixels such that $c\leq i<c_{2}$.

#### Mapping the phosphorylation gradient

To characterise the phosphorylation gradient based on measurements of the phosphorylation at lazy kinetochores, we used a Gaussian process regression approach. A Gaussian process can be seen as generalizing the concept of a multivariate Gaussian distribution from scalars or vectors to functions (Rasmussen and Williams, 2006). Gaussian process regression thus allows us to perform regression over the space of functions. A Gaussian process is completely specified by its mean m(x) and covariance function $k\left(x,x^{\prime }\right)$. Suppose we have *N* inputs, $x_{1},\ldots ,x_{N}\in R$ paired with *N* outputs $y_{1},\ldots ,y_{N}\in R$, then the probability of any finite number of these outputs, *y*, conditioned on the inputs, *x*, is multivariate Gaussian distributed:

y∼N(m(x),K(x|θ))

where m(x) is a vector of length *N*, and K(x|θ) is an N×N covariance matrix. We use a squared exponential kernel for the covariance function:

$$K{\left(x|\alpha ,\rho ,\sigma \right)}_{i,j}=\alpha^{2}\text{exp}\left(-\frac{1}{2\rho^{2}}{\left(x_{i}-x_{j}\right)}^{2}\right)+\delta_{i,j}\sigma^{2},$$

where α,ρ,σ are hyperparameters and $\delta_{i,j}$ is the Kronecker delta taking value 1 if i=j and 0 otherwise. The hyperparameter *α* is the marginal standard deviation controlling the magnitude of the function modelled by the Gaussian process, *ρ* is the length scale which controls the frequency of variation, *σ* is a noise term and ensures positive-definiteness.

Here, the inputs $x_{1},\ldots ,x_{N}$ are distances from the spindle mid-zone in *μ*m and the outputs $y_{1},\ldots ,y_{N}$ are (log-transformed) ratios of KNL1 pS24 signal to CENP-C signal on stretched lazy kinetochores in N=42 fixed cells, which should allow us to infer a function corresponding to the phosphorylation gradient. We apply the log transformation to the outputs since the ratios of KNL1 pS24 signal to CENP-C signal are constrained to be positive. Setting priors for the hyperparameters of ρ∼InvGamma(5,5), α∼N(0,1) and σ∼N(0,1), we infer posterior distributions for these hyperparameters using a MCMC method utilising Hamiltonian Monte Carlo implemented in Stan (Carpenter et al., 2017). Taking hyperparameters as α=1.18,ρ=1.19,σ=0.54 based on the medians of the hyperparameter posterior distribution, we can similarly simulate from the Gaussian process posterior representing the phosphorylation gradient, as shown in Figure 6I.

#### Statistical comparisons

Differences in medians were assessed via two sample Wilcoxon tests. Differences in proportions were assessed via Fisher’s exact test. Correction for multiple testing was performed via the Holm-Bonferroni method. All tests were performed using the rstatix package in the software R v3.5.2.

### Additional resources

None.

## Data Availability

Kinetochore tracking data have been deposited at Zenodo and are publicly available as of the date of publication. All original code has been deposited at GitHub and is publicly available as of the date of publication. DOIs are listed in the key resources table. Any additional information required to reanalyze the data reported in this paper is available from the lead contact upon request.

## Supplemental information

Document S1. Figures S1–S7 and Tables S1 and S2

Document S2. Article plus supplemental information
