# Kinetochore life histories reveal an Aurora-B-dependent error correction mechanism in anaphase

**DOI:** 10.1016/j.devcel.2021.11.023

**Published:** 2021-12-20

**Authors:** Onur Sen, Jonathan U. Harrison, Nigel J. Burroughs, Andrew D. McAinsh

## Main Text

(*Developmental Cell* 56, 3082–3099.e1–e5, November 22, 2021)

Due to an error in Production, the publication date in the online PDF version of this article originally published as November 2, 2021, instead of as the actual November 9, 2021, publication date. This error has now been corrected in the online PDF version of the article. We regret this error and apologize for any confusion that it may have caused.

