# Supplementary material for: Kinetochore life histories reveal an Aurora-B-dependent error correction mechanism in anaphase
Source: Dev Cell. 2021 Nov 22;56(22):3082–3099.e5. doi: 10.1016/j.devcel.2021.10.007 (PMC8629432; doi:10.1016/j.devcel.2021.10.007)
Supplement: Document S1. Figures S1–S7 and Tables S1 and S2 [file mmc1.pdf]

Developmental Cell, Volume *56*

Supplemental information

Kinetochore life histories reveal an Aurora-B-dependent error correction mechanism in anaphase

Onur Sen, Jonathan U. Harrison, Nigel J. Burroughs, and Andrew D. McAinsh

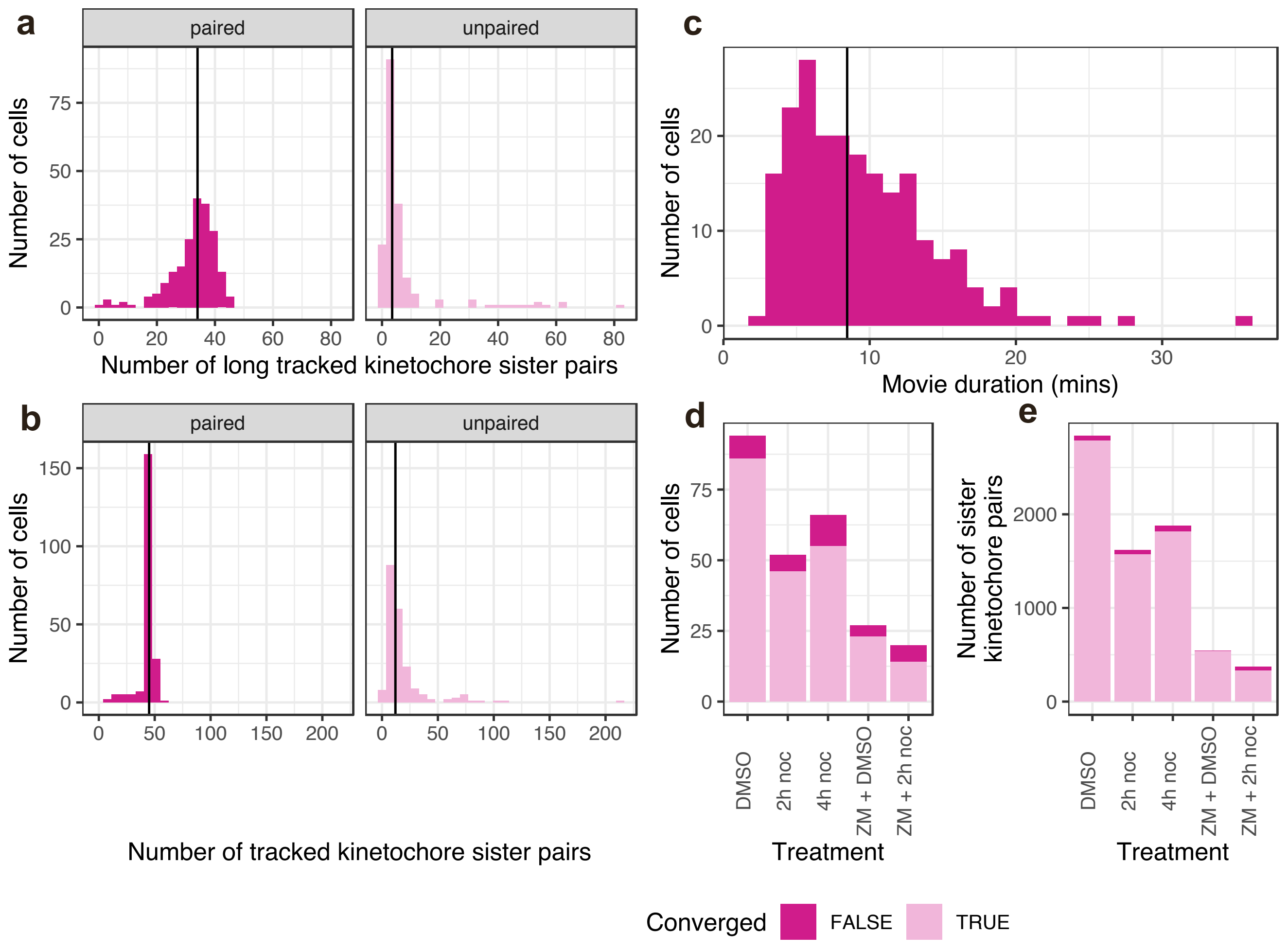

Figure S1. Near complete tracking of kinetochore sister pairs over long timescales. (Related to Figure 1). a, Histograms show the number of cells, in which long tracks (>75% of the 3D time series (movie) for each cell) were obtained for the given number of kinetochore sister pairs (paired) or single kinetochores (unpaired). **b,** Histograms show the number of cells, in which tracks (irrespective of their length in a movie) were obtained for the given number of kinetochore sister pairs (paired) or single kinetochores (unpaired). **c,** Histogram shows the number of cells with the given movie duration. Black lines indicate median in (a), (b) and (c). **d,** Bar chart shows the number of cells from each treatment group for which MCMC chains have converged for the mechanistic anaphase model. **e,** Bar chart shows the number of pairs (within cells that have converged) from each treatment group for which MCMC chains have converged for the mechanistic anaphase model.

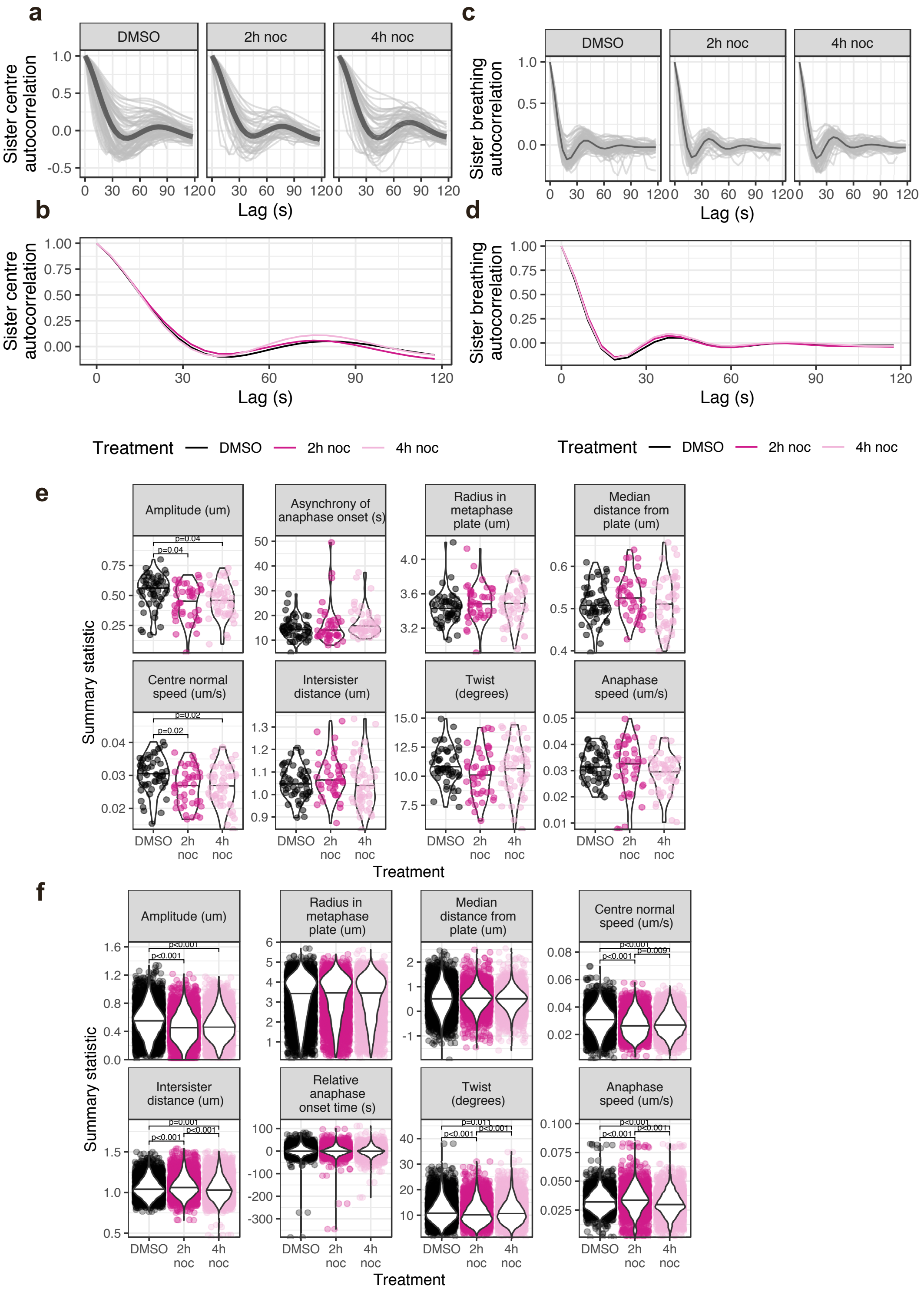

Figure S2. **Nocodazole arrest-and-release has little impact on metaphase oscillation dynamics, except for a reduction in oscillation amplitude. (Related to Figure 1).**

Figure S2. Nocodazole arrest-and-release has little impact on metaphase oscillation dynamics, except for a reduction in oscillation amplitude. (Related to Figure 1). a, Graphs compare the sister centre (mean position of sister kinetochores) autocorrelation for kinetochore oscillations in treatment groups. Each of the light grey curves represents the average (of all kinetochores in that cell) autocorrelation for a cell. Dark grey curve indicates the average autocorrelation for kinetochore oscillations in all cells per treatment group. **b,** Graph shows the average sister centre autocorrelations for the treatment groups plotted together. **c,** Graphs compares the sister breathing autocorrelation for kinetochore pairs in treatment groups, which is a readout for the regularity of breathing movement between sister kinetochores during their oscillations. Each of the light grey curves represents the average (of all kinetochores in that cell) autocorrelation for a cell. Dark grey curve indicates the average autocorrelation for kinetochore breathing in all cells per treatment group. **d,** Graph shows the average sister breathing autocorrelations for the treatment groups plotted together. **e,** Graphs compare eight metaphase-anaphase variables (summary statistics) in the cells (average of kinetochores per cell) treated with DMSO ($N = 53$ cells), 2h nocodazole ($N = 46$ cells) or 4h nocodazole ($N = 54$ cells). Average values are median. **f,** Graphs compare eight metaphase-anaphase variables (summary statistics) in kinetochores from the cells treated with DMSO ($n = 5348$ kinetochores), 2h nocodazole ($n = 4643$ kinetochores) or 4h nocodazole ($n = 5585$ kinetochores). Average values are median.

Table 1. R-squared and p values for significantly correlating variables (Related to Fig. 3)

sumstat	adj.r.squared	p.value
Amplitude (um)	0.07	0.0095
Intersister distance (um)	0.15	<0.001
Centre normal speed (um/s)	0.11	0.0015
Anaphase speed (um/s)	0.02	0.15
Median distance from plate (um)	-0.01	0.76
Radius in metaphase plate (um)	-0.01	0.6
Twist (degrees)	0.08	0.0073
Relative anaphase onset time (s)	0.32	<0.001

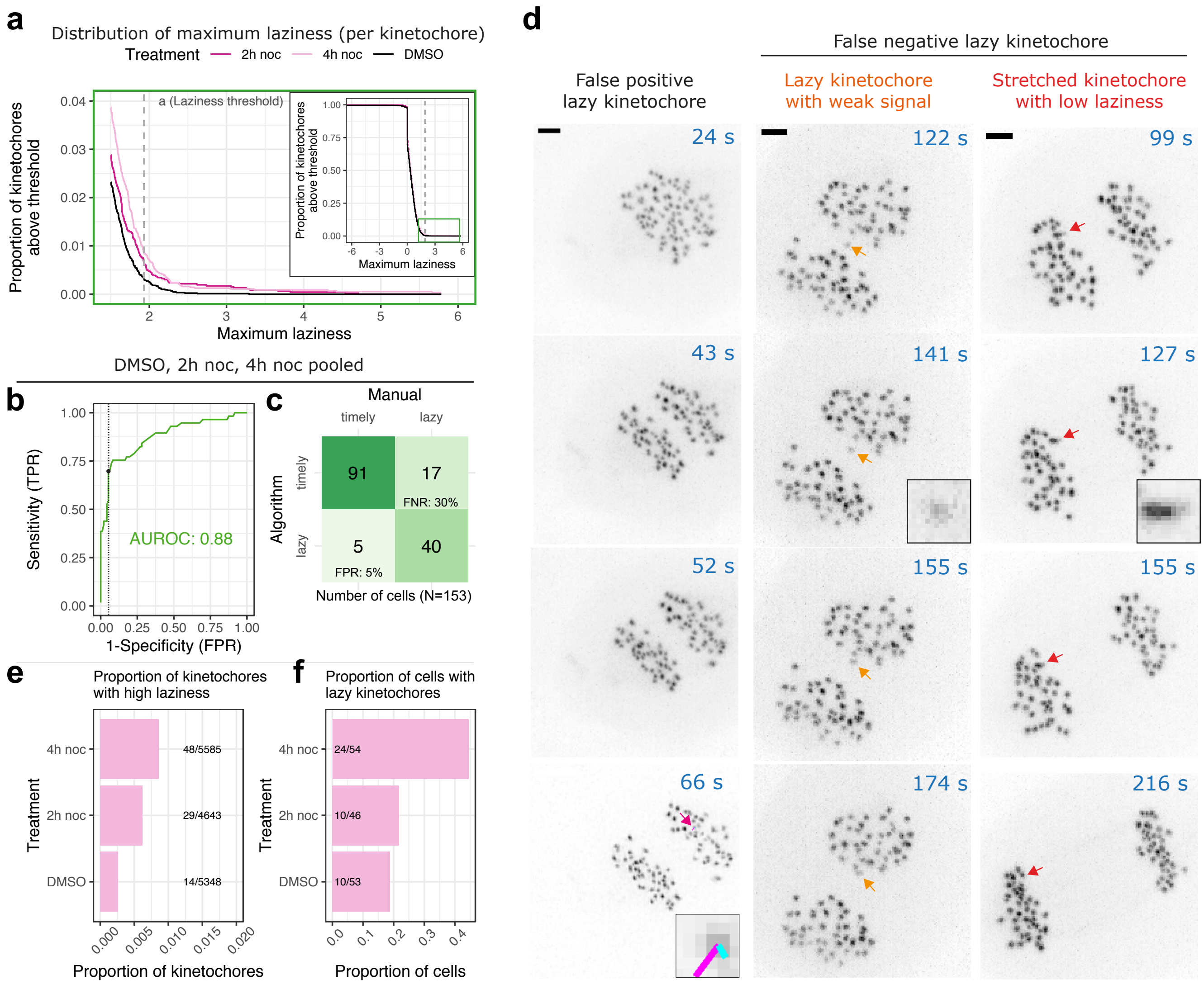

Figure S3. Calibration of the laziness threshold to manual assessment of chromosome segregation. (Related to Figure 2). a, Graph shows the proportion of kinetochores in the population with maximum laziness above a given value, L (from L=1.0 to 6.0) for all kinetochore trajectories from three treatment groups; DMSO, 2h nocodazole, 4h nocodazole. Dashed line denotes laziness threshold. (top-right corner) Graph shows the distribution of all maximum laziness values. **b,** Graph shows receiver operator characteristic (ROC) curve at various laziness threshold settings. The laziness threshold ($a = 1.93$) is denoted by the black dot, and the corresponding false positive rate (5%) is denoted by the dashed line. The area under the ROC curve (AUROC) indicates how good the model is at distinguishing between the cells with at least one lazy kinetochore (reached laziness $> a$ during anaphase) and the cells without lazy kinetochores, (timely). **c,** Confusion matrix demonstrates the number of cells ($N = 153$) having at least one lazy kinetochore (lazy), or zero lazy kinetochores (timely) during anaphase, quantified by the algorithm or manual assessment. False positive rate (FPR) indicates the cells scored as (having) lazy (kinetochore) by the algorithm but not by manual assessment. False negative rate (FNR) indicates the cells scored as lazy (having lazy kinetochore(s)) by manual assessment but not by the algorithm. **d,** Z-projected movie stills of representative cells that (left panel) have a false positive lazy kinetochore annotated with magenta arrow and magenta-cyan dragontails indicated by the algorithm; (middle panel) false negative lazy kinetochore, not detected by the algorithm due to low signal-to-noise ratio (zoomed kinetochore annotated by orange arrow); (right panel) false negative lazy kinetochore, scored as lazy by manual assessment due to merotely indicated by kinetochore stretching, but not by the algorithm due to being close to the cluster centre (zoomed kinetochore annotated by red arrow). Scale bar is 2 μm. **e,** Graph shows the proportion of lazy kinetochores (with max laziness > 1.93 threshold, a) in the cells treated with DMSO, 2h nocodazole or 4h nocodazole prior to washout. Numbers of lazy kinetochores and total kinetochores are shown for each treatment group. **f,** Graph shows the proportion of cells, that have at least one lazy kinetochore, treated with DMSO, 2h nocodazole or 4h nocodazole prior to washout. Numbers of cells with lazy kinetochore(s) and total cells are shown for each treatment group.

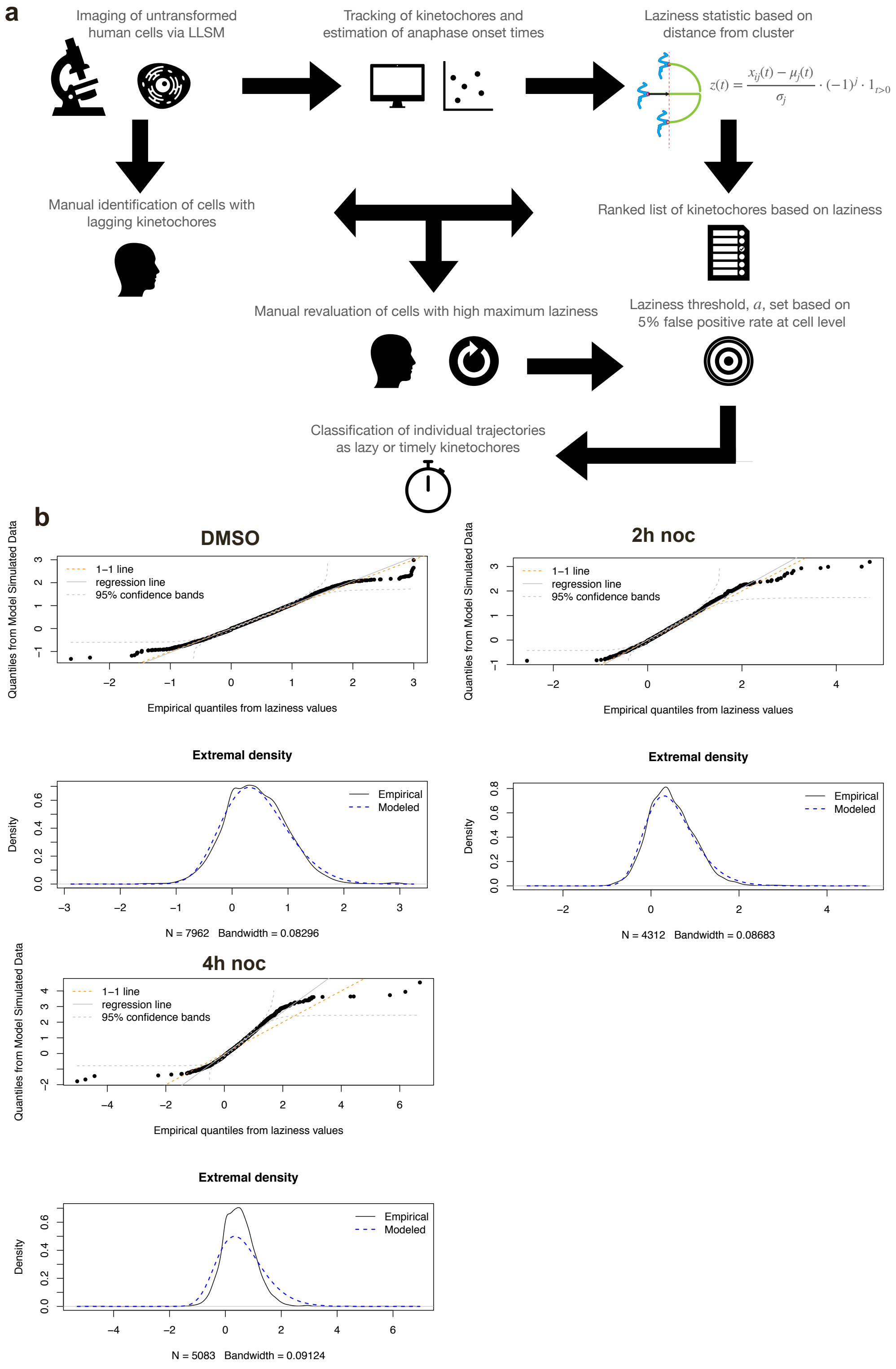

Figure S4. Estimation of laziness threshold and its calibration to manual assessment. (Related to Figure 2). a, Schematic illustrates the data analysis pipeline: data collection (LLSM imaging), tracking of kinetochores and calculation of laziness for each of them, calibration of laziness threshold in the light of manual assessments and automated analyses, and classification of individual kinetochores based on their segregation behavior. **b,** Quantile-quantile (Q-Q) plots show the maximum laziness scores fitted to a generalized extremal value distribution, which suggest that beyond a laziness threshold, a, of approximately 2, the quality of the fit breaks down for kinetochores from the cells treated with DMSO ($n = 5348$ kinetochores), 2h nocodazole ($n = 4643$ kinetochores) or 4h nocodazole ($n = 5585$ kinetochores).

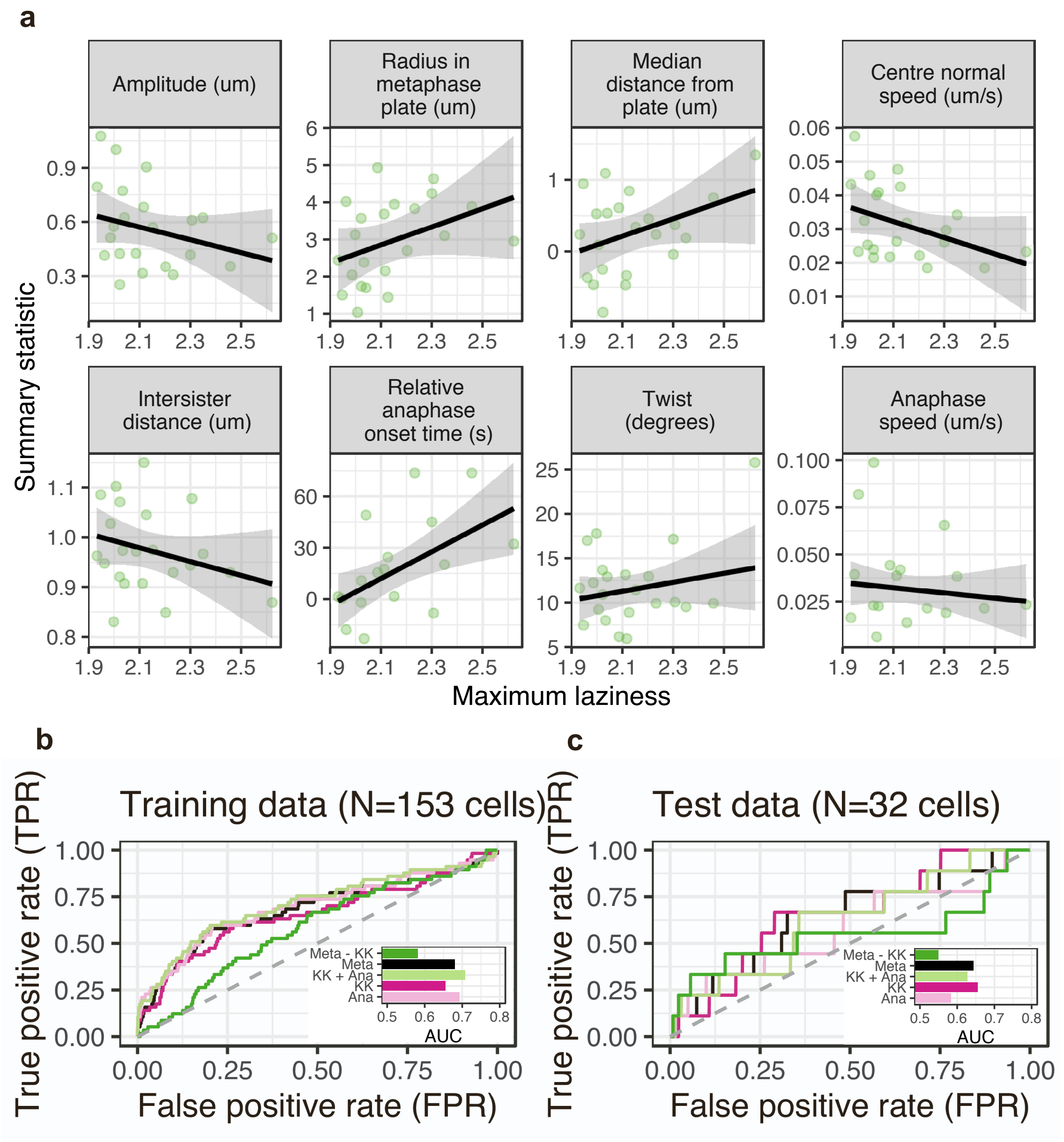

Figure S5. **Metaphase signature is consistent with the lazy kinetochores from unperturbed cells. (Related to Figure 3). a,** Graphs show regression analyses of changes in the eight metaphase-anaphase variables (summary statistics) with respect to the maximum laziness throughout anaphase exhibited by lazy kinetochores from the cells treated with DMSO ($n = 22$ lazy kinetochores). Black lines denote a linear fit to the data obtained via maximum likelihood estimation and the grey envelopes show the 95% confidence interval for predictions. **b,** ROC curve showing predictive capacity of several models on the training dataset ($N = 153$ cells; DMSO ($N = 53$ cells), 2h noc ($N = 46$ cells), 4h noc ($N = 54$ cells)) in classifying lazy kinetochores based on metaphase dynamics. Inset: bar-chart shows the area under the ROC curve (AUC), performance indicator, for each model with colours as for the ROC curves. Covariates used in each model are as follows: Meta: all metaphase variables shown in Fig. 3f; KK: intersister (K-K) distance; Ana: anaphase onset time of a sister pair relative to the median anaphase onset for the cell; Meta - KK: all metaphase variables without K-K distance; Ana + KK: relative time of anaphase onset and K-K distance. **c,** ROC curve showing predictive capacity of several models on a test dataset (DMSO; $N = 32$ cells). Inset: bar-chart shows the area under the ROC curve (AUC) for each model with colours as for the ROC curves; models as (b).

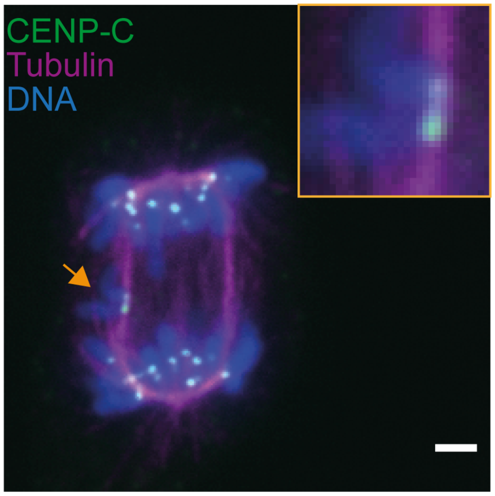

Figure S6. **Stretched lazy kinetochores are attached to microtubules from both spindle poles (merotely). (Related to Figure 4). a,** *Z*-projected image of three adjacent *Z*-planes from a representative cell treated with DMSO (-ZM) for 10 minutes, following 4h nocodazole (0.33 μM) arrest and 40 min washout prior to fixation. Fixed cells were stained with antibodies detecting CENP-C, α-tubulin and with DAPI detecting DNA. Orange arrow indicates a stretched lazy kinetochore attached to microtubules from both poles (merotelic); see inset for enlargement. Scale bar is 2 μm.

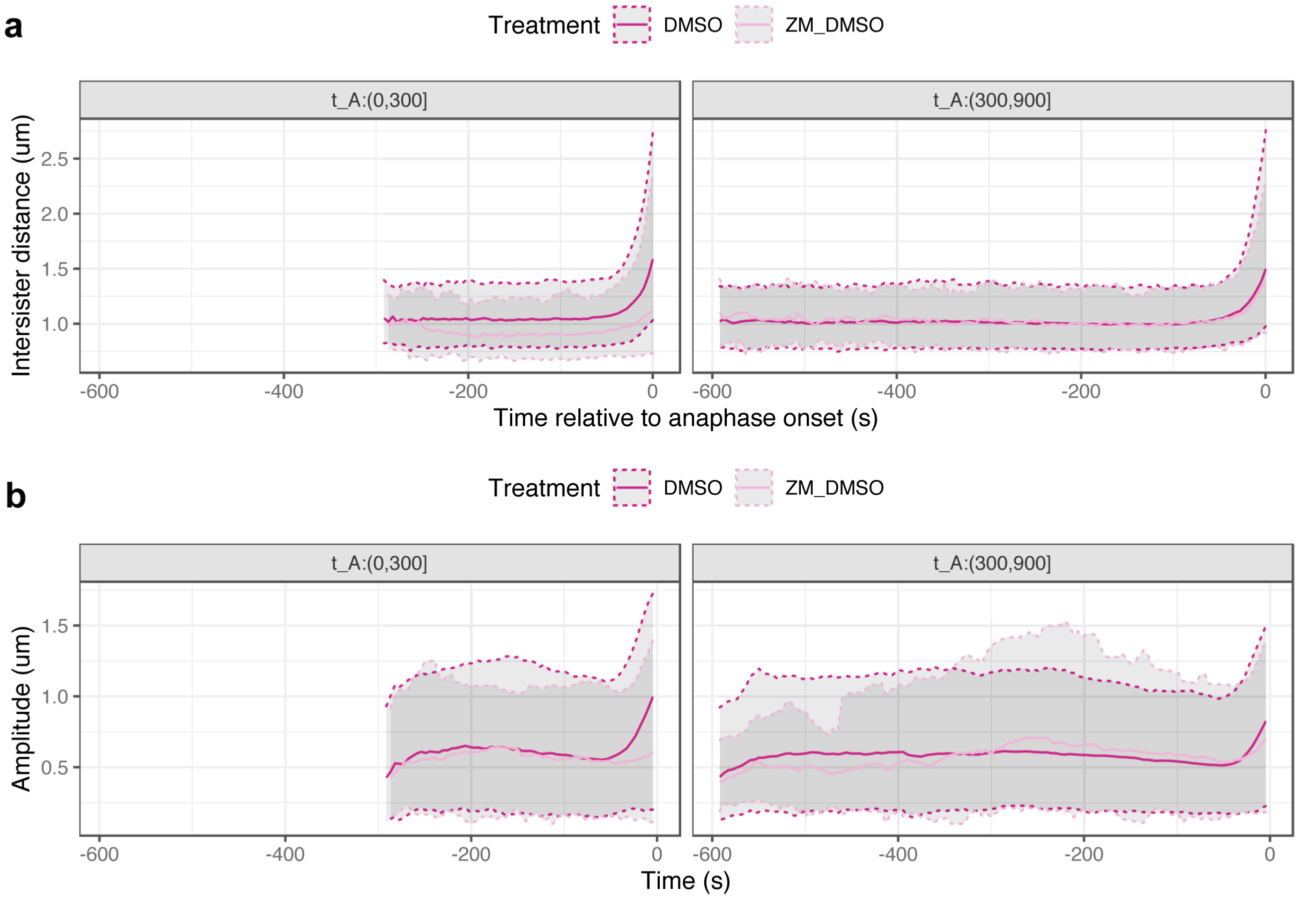

Figure S7. **Aurora B inhibition in fully aligned metaphase cells does not significantly affect metaphase kinetochore dynamics. (Related to Figure 5). a,** Intersister distance over time for cells treated with DMSO (magenta) and ZM + DMSO (pink). The left plot shows cells with a median anaphase onset, t_A, (metaphase duration) less than 300s (DMSO: N=46 cells; ZM+DMSO: N=15 cells) while the right plot shows cells with a median anaphase onset, t_A, (metaphase duration) between 300s - 900s (DMSO: N=40 cells; ZM+DMSO: N=6 cells). The solid line indicates the median over kinetochore pairs, with the dotted lines showing the 2.5% and 97.5% quantiles. **b,** Amplitude over time for cells treated with DMSO (magenta) and ZM + DMSO (pink). The left plot shows cells with a median anaphase onset, t_A, (metaphase duration) less than 300s (DMSO: N=46 cells; ZM+DMSO: N=15 cells) while the right plot shows cells with a median anaphase onset, t_A, (metaphase duration) between 300s - 900s (DMSO: N=40 cells; ZM+DMSO: N=6 cells). The solid line indicates the median over kinetochore pairs, with the dotted lines showing the 2.5% and 97.5% quantiles.

Table 2. **Values for significantly correlating variables under different treatment conditions (Related to Fig. 3).**

treatment	sumstat	value
2h noc	Amplitude (um)	0.451
4h noc	Amplitude (um)	0.462
DMSO	Amplitude (um)	0.555
2h noc	Radius in metaphase plate (um)	3.47
4h noc	Radius in metaphase plate (um)	3.47
DMSO	Radius in metaphase plate (um)	3.37
2h noc	Median distance from plate (um)	0.533
4h noc	Median distance from plate (um)	0.518
DMSO	Median distance from plate (um)	0.506
2h noc	Centre normal speed (um/s)	0.0264
4h noc	Centre normal speed (um/s)	0.0270
DMSO	Centre normal speed (um/s)	0.0315
2h noc	Intersister distance (um)	1.06
4h noc	Intersister distance (um)	1.03
DMSO	Intersister distance (um)	1.04
2h noc	Relative anaphase onset time (s)	0.00
4h noc	Relative anaphase onset time (s)	0.00
DMSO	Relative anaphase onset time (s)	0.00
2h noc	Twist (degrees)	10.1
4h noc	Twist (degrees)	10.7
DMSO	Twist (degrees)	11.1
2h noc	Anaphase speed (um/s)	0.0338
4h noc	Anaphase speed (um/s)	0.0297
DMSO	Anaphase speed (um/s)	0.0343
